# Chemotaxonomic Evaluation by Volatolomics Analysis of Fifty-Two Genotypes of *Myrtus communis* L.

**DOI:** 10.3390/plants9101288

**Published:** 2020-09-29

**Authors:** Marianna Usai, Mauro Marchetti, Nicola Culeddu, Maurizio Mulas

**Affiliations:** 1 Department of Chemistry and Pharmacy, University of Sassari, Via Muroni 23/A, I-07100 Sassari, Italy; 2 Institute of Biomolecular Chemistry, National Research Council (CNR), Trav. La Crucca 3, 07100 Sassari, Italy; mauro.marchetti@cnr.it (M.M.); nicola.culeddu@cnr.it (N.C.); 3 Department of Agriculture, University of Sassari, Via De Nicola 9, I-07100 Sassari, Italy

**Keywords:** *Myrtaceae*, Myrtle leaves, volatile composition, GC/MS, statistical analysis

## Abstract

A population of 52 genotypes of Myrtle (*Myrtus communis* L.), selected in the framework of a domestication program and growing in the same collection field at Oristano (Central Western Sardinia, 39°54′ N 8°35′ E), was analyzed by GC/MS for leaf essential oil composition. The chemical composition of essential oils was quite variable with a number of compounds ranging from 31 to 78 depending on cultivar. One hundred and eighteen compounds were globally identified in the various genotypes. However, α-pinene, limonene, 1,8-cineole, α-terpineol, and linalool always resulted as main components with few differences among samples. Minor compounds have been the determining factors in differentiating or associating genotypes in the outputs of a principal component analysis (PCA), where the results of another analysis of fruit essential oils of the same genotypes were also jointly used. Genotypes were discriminated according to mother plant characterization or ecological variables, such as site altitude, soil nature, and presence or absence of calcareous soils in the substrate of the localities of origin.

## 1. Introduction

*Myrtus communis* L. (Myrtle) is a wild aromatic plant that belongs to the Myrtaceae family. This medicinal species is native across the Mediterranean region, Macaronesia, Western Asia, and the Indian subcontinent. It is the only European genus of the Myrtaceae family, which largely spreads in tropical and subtropical areas, and it is cultivated in gardens in Mediterranean-type climates, due to its fragrant flowers, although the leaves and berries are also scented [1]. Myrtle is a diploid drought-tolerant evergreen shrub, 0.5–3.0 m in height, which branches from a close canopy, thickly covered with 3–5-cm-long ovate or lanceolate leaves, and can be found in damp or sunny places up to ≈800 m a.s.l. [2].

A characteristic Myrtle liqueur is one of the main typical productions from Sardinia. Some parts of the plant are used in the food industry for flavoring meat and sauces, and its leaves have been extensively used in perfume and cosmetic industries [3,4]. Myrtle has also been intensively studied for its essential oil obtained from the whole aerial part of the plant [5], leaves [3,6], flowers [3], and berries [4]. Different chemical profiles according to different plant parts sampled, or different test populations, have been registered. Normally, the main compounds in leaf and flower essential oils are α-pinene, linalool, and 1,8-cineole, while myrtenyl acetate is often the main compound in white berry essential oil [7,8,9,10]. 

**Figure 1:**
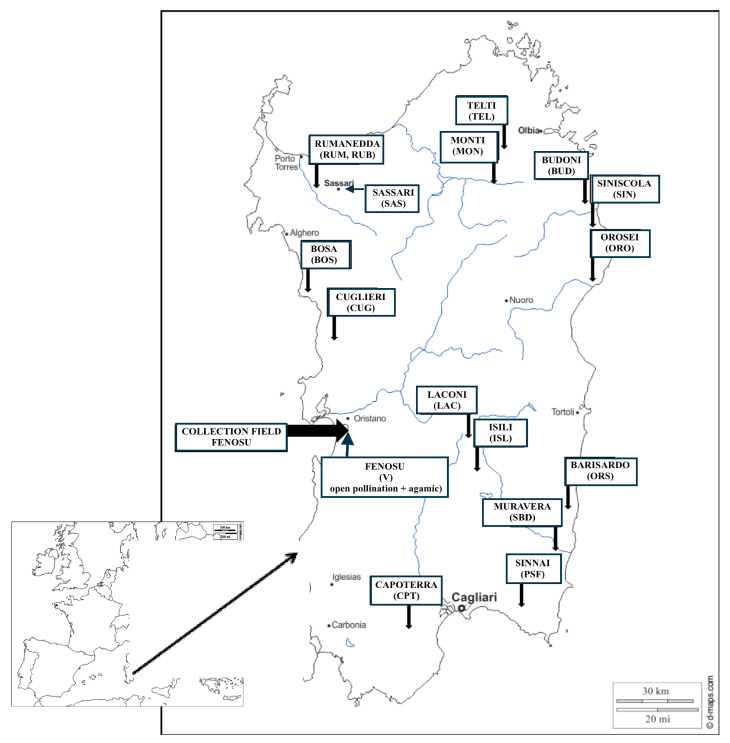
Field location of Fenosu (Oristano) and localities of Myrtle selections with the acronyms of samples investigated in the present work.

The chemical components of the essential oils obtained from all tissues of the plant determine the antioxidant, antibiotic, and antimutagenic properties of the Myrtle biomass [11,12,13]. Several studies have indicated the activities of Myrtle chemical components [14,15]. It is known that intraspecific variability and environmental conditions can significantly influence the qualitative and quantitative levels of major compounds of essential oils that characterize a plant species. Based on the aim of selecting a commercially exploitable cultivar, considerable effort has been dedicated to collecting wild plants (with good qualitative and quantitative production) around all Sardinia, and to their agamic propagation. Candidate clone-lines in this first mass-selection step were planted in a comparison field, where the best genotypes were described, tested as cultivars, and proposed to the nursery market [16,17]. Another selection group (named “V” + code number) was obtained by open pollination of some previously selected candidate clones, sowing of the hybrids, transplanting a lot of seedlings in a plot, and after some years, the selection of new candidate clones by agamic propagation in a new plot (Table 1). All these selections are still cultivated in the Fenosu experimental field and represent a wide population we can use to investigate chemical variations in these genotypes (Figure 1, Table 1). In this field, the production of different chemical profiles, in all parts of these plants, is regulated only by genetic differences because environmental conditions are the same for all populations. 

In this study, we investigated the intra-specific variability of essential oil compositions in a population of 52 *M. communis* L. selections in order to characterize the chemical profiles among different samples of the same species in the population. The purpose was to test whether the intra-specific genetic variability of this species was influenced by geographic, soil, and/or environmental factors fixed in the mother plants from which the genotype collection was derived. 

To achieve this aim, a chemometric method was applied. For this purpose, the experimental data were analyzed using Principal Component Analysis (PCA) and orthogonal projection methods, in particular Orthogonal Partial Latent Structure Discriminant Analysis (OPLS-DA), which is a highly accurate statistical method and is a powerful modelling tool. It provides insights into the separations between experimental groups, based on high-dimensional spectral measurements obtained with NMR, MS, or other analytical instrumentation [18]. In particular, in OPLS, continuous variable data were separated into predictive and unrelated information. This allows one to improve and facilitate diagnostic interpretations and obtain a more intuitive visualization [19]. However, these changes only improve the interpretability, not predictability, of Partial Least-Squares regression (PLS) models. The best is the OPLS-DA (Discriminant Analysis) method that can be applied when working with discrete variables, such as in classification and biomarker studies. In OPLS-DA, objects are classified into group clusters with predetermined models for the class. This is an excellent tool for finding "What is the difference" between two groups (like Good and Bad products). The OPLS-DA model indicates the driving forces between variables. Therefore, we can create graphs (score plots) to visualize differences if these exist. We can also use graphics that can display variables that express this difference (loading plots).

## 2. Results and Discussion

In our studies, we considered the differences in essential oils from the leaves of 52 different candidate clones collected from accessions of *M. communis* L. preserved in a collection field at the University of Sassari (Fenosu). 

**Figure 2:**
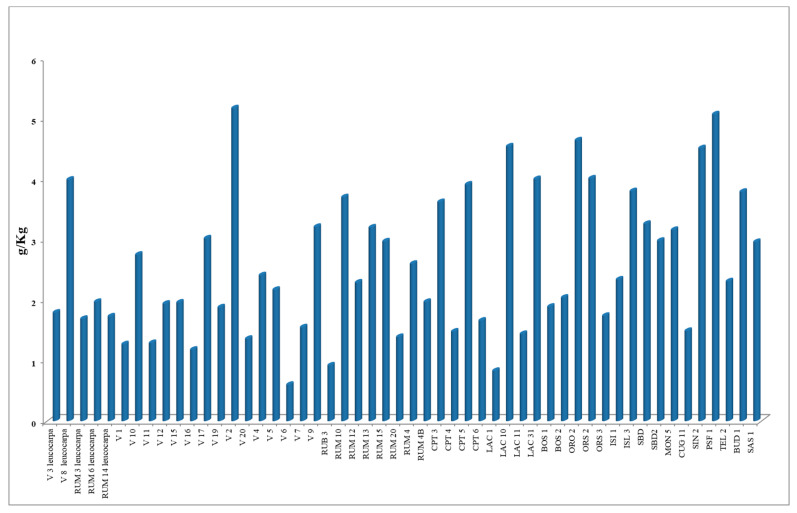
Essential oil yields in leaves of Myrtle accessions (LSD = 0.69 g·kg^−1^ at *p* ≤ 0.01).

The selections are represented by five cultivars of var. *leucocarpa* and 47 cultivars of var. *melanocarpa.* GC/MS analyses permitted us to identify a total of 118 compounds, even if not all were present in every selection (Tables S1–S5); the essential oil yields were significantly variable (Figure 2).

Looking at the *leucocarpa* variety, selection RUM3 reached the minimum (1.69 g·kg^−1^) and V8 the maximum (3.99 g·kg^−1^) oil yield. In other samples, the essential oil yield was below 2.00 g·kg^−1^. In the *melanocarpa* variety, in selections named as “V” series, the minimum yield was found in V6 (0.60 g·kg^−1^), and the maximum yield of the analyzed essential oils was in V2 (5.17 g·kg^−1^). Other good yields were found in LAC (4.00 g·kg^−1^ and more) and in PSF1 (5.00 g·kg^−1^).

GC/MS analysis of the *leucocarpa* variety (first five accessions in Table S1) showed a wide variability, and the main components (Table 2) were α-pinene, limonene, and 1,8-cineole. In RUM14, α-pinene reached a high concentration, and the lowest was in V8, while limonene was characterized in V3 and V8 selections with high concentrations. The majority of 1,8-cineole was concentrated in V8 and RUM6. It is interesting to note that linalyl acetate was present only in RUM14.

The *melanocarpa* variety (Table 2, Table 3, Table 4, Table 5 and Table 6) was most widespread than *leucocarpa*. In the RUM selection, α-pinene reached the maximum in RUM10, different from other selections that showed concentrations varying from 30% to 17%, and limonene and 1,8-cineole were always present (Table 2).

The 15 selections labelled as “V” were characterized by 74 different constituents, distributed in a very different way, and the major constituents were α-pinene, limonene, 1,8-cineole, and linalool (Table 3 and Table 4).

Linalyl acetate was found only in V15 and V10, and myrtenyl acetate was found in some accessions in very low concentrations, except in V15. Dihydroeugenyl butanoate was present in V17 and V2. On the other hand, linalyl acetate was present only in two selections, RUM15 and RUM20, and dihydroeugenyl butanoate reached the maximum in RUM12.

Table 5 evidences high concentrations of isobutyl isobutyrate, 1,8-cineole, and geranyl acetate in the CPT3 accession. The discriminating compound in this selection was α-terpinyl acetate, present only in CPT3. In the Laconi (LAC) accession the presence of p-cymenene was recognized in LAC11, which was not detected in other accessions. In the LAC10 clone, 10.63% of dihydroeugenyl butanoate was detected. 

In the ORS selection (Barisardo), coming from the East cost of Sardinia, ORS2 was recognizable by the major concentrations of limonene, 1,8-cineole, α-terpineol, geranyl acetate, and dihydroeugenyl butanoate; while ORS3 showed the highest concentration of α-pinene. In the two BOS selections, we found a distinctive compound, myrtenyl acetate. In ISL (Isili) selections, in spite of the common origin from the same geographical area, they were quite different each other. In fact, in ISL1 we found o-cymene and *Cis*-geraniol, which were not present in ISL3.

In SBD (Muravera) selections, SBD1 was identifiable by the higher presence of dihydroeugenyl butanoate. Other differences were due to presence of methyleugenol and linalool in SBD1 (Table 6).

In addition to previously presented results, eight more selections (MON5, CUG11, SIN2, TEL10, BUD1, PSF1, and SAS1) were represented by only one sample (Table 6). In the MON5 selection, major constituents were α-pinene, 1,8-cineole, limonene, and geranyl acetate. In CUG11, as principal constituents are concerned, a similar concentration as MON5 were mostly found. In PFS1 a high concentration of dihydroeugenyl butanoate was observed. 

SAS1 is a selection of var. *tarantina,* and the chromatographic profile of this clone, as expected, was very different from that of the other selections. It was characterized by myrtenyl acetate (28.13%) as the principal constituent, with a high content of limonene and a low content of α-pinene.

All data on the essential oils analysis in leaves have been reported in Tables S1–S5. The study widely confirmed results from previous research, with α-pinene, 1,8-cineole, limonene, linalool, and α-terpineole as the main compounds characterized in Myrtle essential oils of Sardinia [20,21]. This composition was substantially similar to those of essential oils from Tunisia, Corsica, and Liguria [3,8,13,22,23], while the low concentrations of myrtenol and myrtenyl acetate were confirmed as discriminant with respect to samples coming from the East and West Mediterranean areas [13,23,24]. The importance in some genotypes of two previously identified compounds, dihydroeugenyl butanoate and dihydroeugenyl pentanoate, was also confirmed [4].

The essential oils of leaves were more homogeneous compared to those derived from berries. In order to investigate statistically possible ecological influences on genotype differences and consequent essential oils compositions, we compared data (obtained using the same analytical methods) on the composition of essential oils from leaves and berries obtained from the same selections [4]. The software employed was “SIMCA” (see Materials and Methods), and we used a method based on PCA for classification and OPLS-DA for discriminatory analysis.

A first check on classifiers confirmed the validity of choices. In fact, only a very limited number of these were in a not significant range. A simple analysis of the main components found no homogeneous accession groups (classifiers).

To check whether there were any differences in variables to classify samples and to create predictive models, we used OPLS-DA (Discriminant Analysis) analysis. Consequently, we tried to correlate the compositions of essential oils, derived from berries and leaves of accessions, with discrete variables such as the place of origin, mean rainfall per year, or to plant berry yield and plant vegetative vigor of the mother plant.

**Figure 3:**
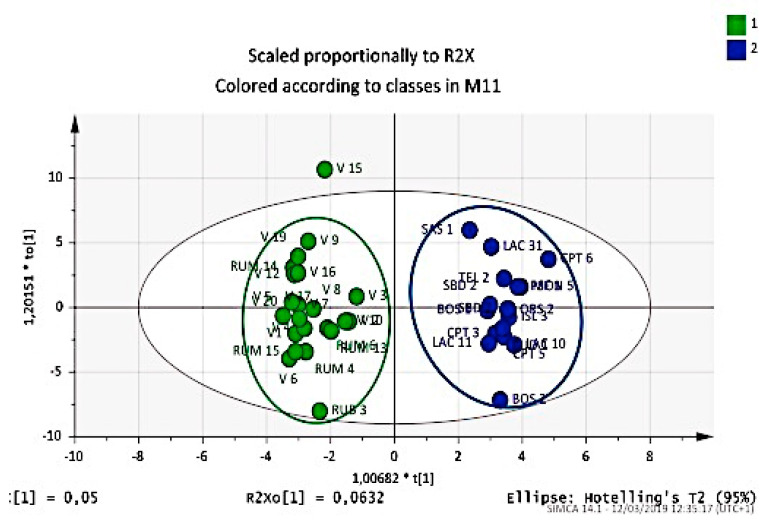
OPLS-DA score plot of the essential oil components from leaves and berries for classification of Myrtle selections correlated with the native area. Rumanedda (green) *vs* other Sardinian accessions (blue).

The first attempt was to relate the place of origin. With this aim, we tried to verify if the composition of essential oils could discriminate between the large group from Rumanedda and cultivars coming from free pollination, with respect to those coming from other parts of Sardinia. The result was surprising, as can be seen from the score plot shown in Figure 3.

This clear division into two groups led us to investigate the reason for this difference. One of the soundest reasons is that accessions from Rumanedda belong to very ancient and virgin Mediterranean flora on which anthropic pressure has not been applied. This has allowed the ecosystem to conserve a marked biodiversity so that plants from Myrtle have the opportunity to diversify into numerous genotypes. Moreover, this group was extensively used as the mother genotypes in the open pollination test, which produced new hybrid selections of the V group. This reflects on the composition of essential oils, and discriminatory analysis clearly highlights the groups Rumanedda and V as compared to all other accessions coming from the territory of Sardinia.

To evaluate the predictive capacity, the generated model was validated by the leave-one-out cross-validation method. As a result, the predictive ability of these models (percentage of the objects belonging to the testing set correctly classified using the developed model) was >97.7%, which revealed that this model was robust and permitted us to recognize differences arising in compositions from different places, rainfall, soil nature, ecosystem nature, fruit production, and plant vigor.

The discriminant functions achieved a good recognition ability (percentage of the objects belonging to the training set correctly classified) of 97.9%, based on the oil constituent concentrations. 

**Figure 4:**
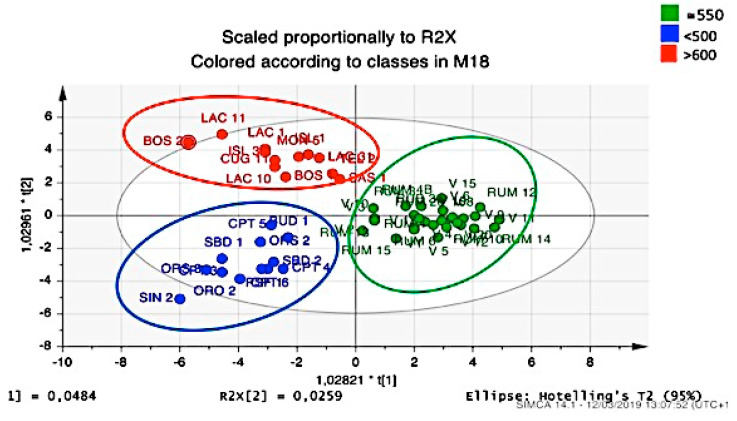
OPLS-DA score plot of the essential oil components from leaves and berries for classification of Myrtle selections correlated with mean annual rainfall of the native area: Rumanedda (green); localities <500 mm rain/year (blue); localities >500 mm rain/year (red).

Furthermore, we considered territorial surveys noted during original plant collections from various stations. In the first instance, the average rainfall detected by the Sardinian meteorological system was considered. Averages are representative for a period of about 100 years; values of rainfall were available from 1922 to today. Figure 4 shows a clear division into three representative accession groups.

In this case, we can easily observe that, once again, Rumanedda and V selections constituted a homogeneous group for the same reasons described above. However, rainfall was a significant discriminant for other accessions. In fact, the composition of essential oil was reminiscent of the average rainfall in the area of origin. In this way, taking advantage of the predictive model, it is possible to choose a collecting area of wild plants to be agamically propagated with the aim to obtain essential oils with desired composition in cultivated plants.

**Figure 5:**
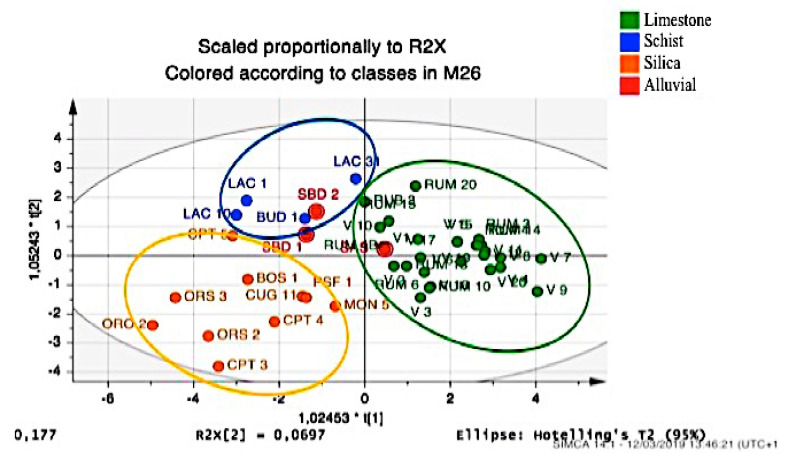
OPLS-DA score plot of the essential oil components from leaves and berries for classification of Myrtle selections correlated with soil type of the native area: limestone (green); schist (blue); silica (orange); alluvial (red).

A similar analysis was also carried out considering the geological nature of soil. Table 7 shows soil origin types for the analyzed accessions, and Figure 5 shows the relative score plot.

The selections based on alluvial soil were positioned close to the prevailing composition of substrate. Consequently, accessions SBD2 and SBD1 coming from Muravera were very close to those coming from schist substrates and to those coming from Budoni (BUD), where soil has a schist nature, but were not very far in composition from the alluvial soil located further South along the same Eastern coast of Sardinia. The SAS selection, which comes from an alluvial soil near Sassari, but is native to Apulia where calcareous land is prevalent, showed a particular positioning. In this case, the model in relation to soil type can also predict the composition of essential oil from where the original plant was collected.

**Figure 6:**
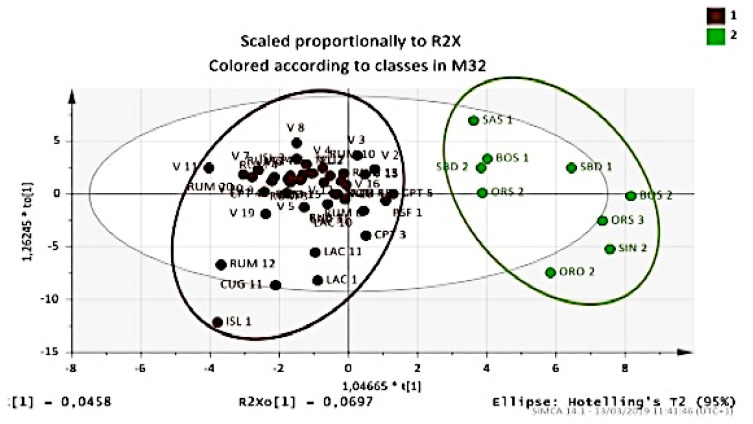
OPLS-DA score plot of the essential oil components from leaves and berries for classification of Myrtle selections correlated with ecosystem type: Mediterranean bush or wood (brown); arable land or pasture (green).

Essential oil compositions were also influenced by the surrounding environment (Ecosystem) from where genotypes originated. In fact, we found a significant differentiation between accessions collected from uncultivated land (Mediterranean maquis or wood) and those deriving from arable land or pastures (Figure 6). A “memory” effect in plants was evident and significantly influenced the composition of essential oil, which allowed us to construct a predictive model.

**Figure 7:**
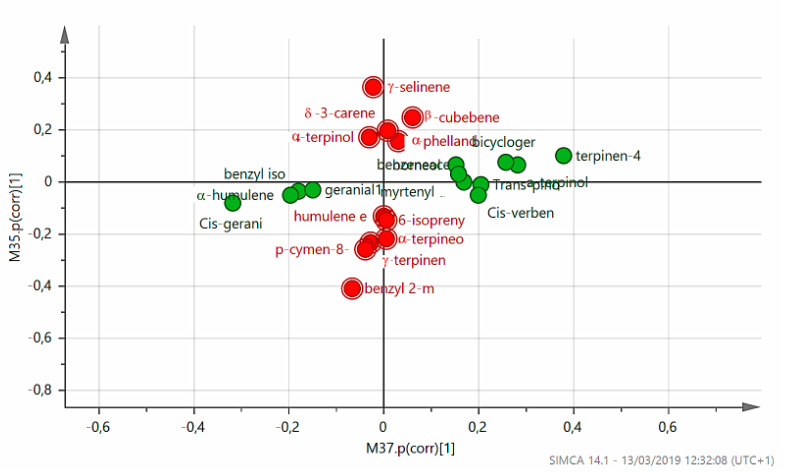
Comparison between correlation coefficients of the models M26 (Figure 5) and M32 (Figure 6).

Comparison of the last two statistical analyses made it possible, based on the model, to establish variables that best characterized accessions, in relation to the land from which they were collected and to the ecosystem in which they evolved over time. Figure 7 shows a comparison between the two models mentioned above, the “land” model (Figure 5) and “ecosystem” model (Figure 6), taking into account the correlation coefficients Pcor. If the ratio between variables in the two models is >3, a particular significance for the higher Pcor model is suggested.

**Figure 8:**
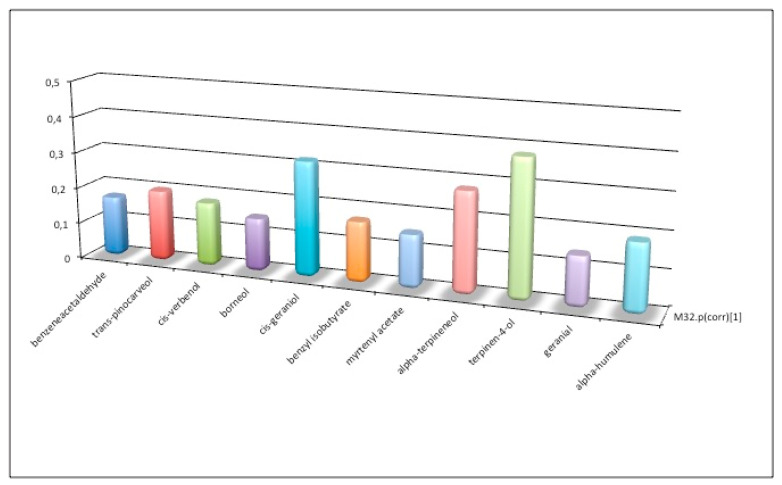
Components of essential oils influenced by soil type. When coefficients tend to zero for the land model (calcareous, siliceous, schist, or alluvial), other terpenes are highlighted (Figure 8). The “Mediterranean maquis” ecosystem stimulates emanation of aromas and odors that are able to attract insects or other organisms, which can carry pollen from other plants, or nursing animals that help to disperse seeds over a wide area in a very competitive environment full of fragrant wild flowers (Figure 9).

From Figure 7 we can observe that if coefficients tend to zero for the ecosystem model (which comprises Mediterranean bush, arable land, or pasture), it is possible to identify which variables depend significantly on land type where genotypes developed (Figure 5). In this case, some terpenes in essential oils play a role. In fact, these terpenes help plants to survive at very high temperatures and lack of water, protecting the photosynthesis apparatus from excess radiation, and contributing to the plant’s adaptation to environmental stresses; moreover, terpenes may protect against pest attacks (Figure 8).

The last variables we used for our discriminant analysis were plant fruit yield and vegetative vigor.

We assigned discrete values for these two variables:

− vigor: (1) medium-poor; (2) medium-low; (3) medium; (4) medium-high; (5) high;
− fruit yield: (1) medium-low; (2) medium; (3) medium-high; (4) high.

**Figure 10:**
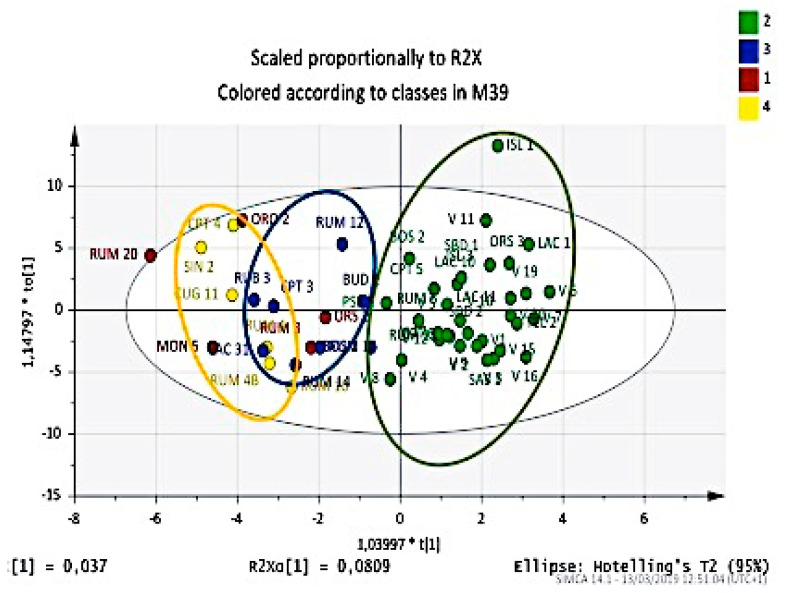
OPLS-DA score plot of the essential oil components from leaves and berries for classification of Myrtle selections correlated with fruit production. Medium-low (red), medium (green), medium-high (blue), high (yellow).

**Figure 11:**
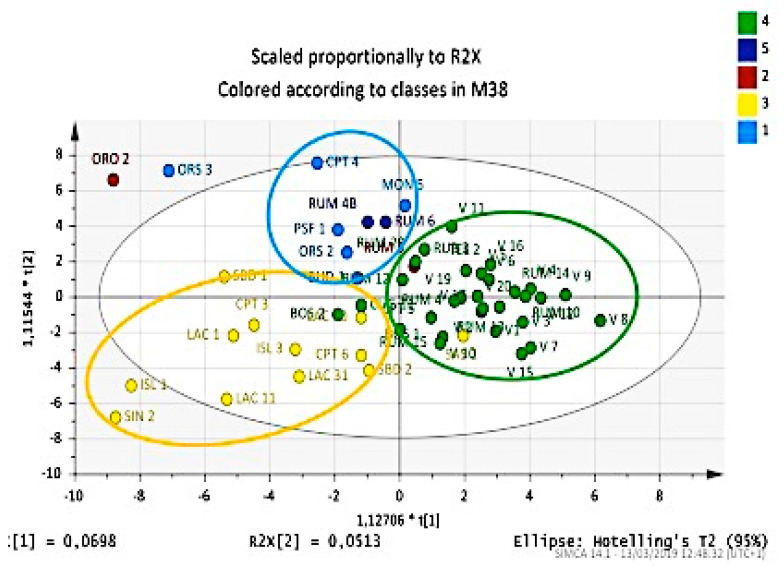
OPLS-DA score plot of the essential oil components from leaves and berries for classification of Myrtle selections correlated with plant vigor. Medium-poor (light blue), medium-low (red), medium (yellow), medium-high (green), high (blue).

Score plots in Figure 10 and Figure 11 show the relative OPLS-DA. The score plot related to the OPSL-DA was calculated using a productivity variable as a discriminant. It is possible to observe a division of accessions into three distinct groups, even if not well defined. The group of plants with average productivity was very well defined, which was also the most representative numerically. Groups with medium-high and high productions were quite distinct from each other, even if there were approaches and intersections between a few accessions, while the low-productivity group diffused randomly on the left part of score plot (Figure 10).

The score plot related to the discrete variable “vigor” shows how accessions gathered into three distinct groups, even if the three groups exclusively concerned vigorous plants: medium-high, medium, and low (which includes the medium-poor categories and medium-low). With regard to accessions that have a high vigor (there were only two), ORO2 and RUM3 did not group together and were randomly distributed in the score plot. 

The results obtained from these two models that considered 217 variables (corresponding to compounds identified in the analyzed essential oils) perfectly match the point observations of the macroscopic productivity and vigor data (Figure 11).

The usefulness of the two models, reported in Figure 10 and Figure 11, respectively, is appreciable if considered together. In fact, from the composition of essential oils of each cultivar shown in the two score plots, it is possible to choose, for example, a plant with low vigor and high productivity. In this way, it would be easier to characterize less demanding cultivars from an agronomic point of view and find those with generous yields, even without knowing these characteristics beforehand. Therefore, by conducting an analysis of essential oils on a spontaneous Myrtle plant, it will be possible, by applying the model, to predict the vigor and fruit productivity and then decide whether it will be convenient or not.

## 3. Conclusions

Application of the orthogonal partial Latent Structure Discriminant Analysis (OPLS-DA) method to analytical data has proven to be very effective in constructing predictive models for Myrtle plants. Our results may contribute to understand the variability and quality of essential oils of this species, indicate the ideal places to collect wild plants, and devote to the cultivation and industrial production of essences and liqueurs. Furthermore, it is possible to have valid indications of the vigor and productivity of selected plants without having to carry out long-term cultivation experiments.

## 4. Material and Methods

### 4.1. Plant Materials and Essential Oils Distillation

The leaves were harvested from the experimental farm “Antonio Milella” located in San Quirico (Fenosu-Oristano, Central Western Sardinia, Italy: 39°53′, 8°37′ E, 12 m a.s.l.) in July 2015 without flower and fruits. Among the 52 cultivars, 5 belonged to the variety *leucocarpa* DC, and 47 belonged to the variety *melanocarpa* DC. Selections originated from different localities of Sardinia [18]. At least 15 plants were represented in every cultivar. Mulas M. identified the analyzed plants. Voucher specimens have been deposited at the Herbarium SASSA (Sassari) of the Department of Chemistry and Pharmacy, University of Sassari, under collective number 514. The description of samples is reported in Table 1 (see introduction).

To avoid harvesting an unrepresentative sample, we collected leaves from the entire plant, collecting material from the top, from the sides, and from the base in triplicate. In the laboratory, the plant material was cleaned of foreign parts (little branches, unripe fruits, residues of flowers), and samples were made as uniform as possible. From every cultivar, about 3 kg of leaves was collected and divided into three parts to replicate analyses. After harvest, clean leaves were kept in a freezer at −20 °C until extraction. Essential oil samples were obtained from leaves via hydrodistillation for 4 h using a Clevenger-type apparatus. For every selection three extractions were performed. Extraction yields were calculated as g·kg^−1^ of fresh material. Oils were stored in sealed vials, at −20 °C, until chemical analysis. 

### 4.2. Gas Chromatography–Mass Spectrometry (GC/MS) Analysis

***GC.*** Three replicates of each sample were analyzed by using a Hewlett-Packard Model 5890A GC, equipped with a flame ionization detector and fitted with a 60 m × 0.25 mm (I.D.), thickness 0.25 μm ZB-5 fused silica capillary column (Phenomenex, Torrance CA, USA). The injection port and detector temperatures were maintained at 280 °C. The column temperature was programmed from 50 °C to 135 °C at 5 °C/min (1 min), 5 °C/min up to 225 °C (5 min), 5 °C/min up to 260 °C, and then held for 10 min. Samples of 0.2 μL (volume injection) were analyzed and diluted in hexane using 2,6-dimethylphenol as internal standard. Injection was undertaken using a split/splitless HP 7673 automatic injector and helium as carrier gas. Several measurements of peak area were performed with a HP workstation with a threshold set to 0 and peak width 0.02. Quantization of each compound was expressed as absolute weight percentage using the internal standard and response factors (RFs). The detector RFs were determined for key components relative to 2,6-dimethylphenol and assigned to other components based on functional group and/or structural similarity, since oxygenated compounds have a lower detectability by FID (Flame Ionization Detector) than hydrocarbons. Standards (Sigma-Aldrich, Fluka and Merck grade) were >95%, and the actual purity was checked by GC. Several response factor solutions were prepared that consisted of only four or five components (plus 2,6-dimethylphenol) to prevent interference from trace impurities. It is known that oxygenated compounds have a lower sensitivity than hydrocarbons to FID. We calculated the response factor using a standard mixture of α-pinene, α-terpineol, nerol, geranial, geranyl acetate, and caryophyllene. The mixture accounted terpenes for 92%, aldehydes 5%, and alcohols, esters, and sesquiterpenes 1% each. In our analyses we obtained a hydrocarbon RF equal to 1, while for alcohols it was 0.80, and for esters 0.71. For this reason we multiplied experimental data with the following correction factors: 1 for hydrocarbons, 1.24 for aldehydes and ketones, 1.28 for alcohols, and 1.408 for esters.

***GC/MS***. MS analyses were carried out with an Agilent Technologies model 7820A connected to an MS detector 5977E MSD (Agilent), using the same conditions and column described above. The column was connected to a mass spectrometer ion source. Mass units were monitored from 10 to 900 AMU at 70 eV. For the identification procedure we considered only peaks from 40 to 900 AMU. Identification of constituents was based on comparisons of Kovat’s index values and mass spectra with those obtained from the authentic samples and/or Nist and Wiley library spectra, or based on interpretation of the EI fragmentation of the molecules [25,26].

### 4.3. Statistical Analysis 

Oil yield data were processed via ANOVA by means of MSTAT-C software, and mean separation was performed by application of Tukey’s test with *p* ≤ 0.05 level of significance. Data were submitted to multivariate statistical evaluation. Prior to chemometric analysis, we set the total integral areas to 100 to normalize the data, and the generated ASCII file was imported into Microsoft EXCEL to add labels. The matrix was imported into SIMCA-P software version 12.0 (Umetrics AB, Umeå, Sweden) for statistical analysis [27].

## Figures and Tables

**Figure 9:**
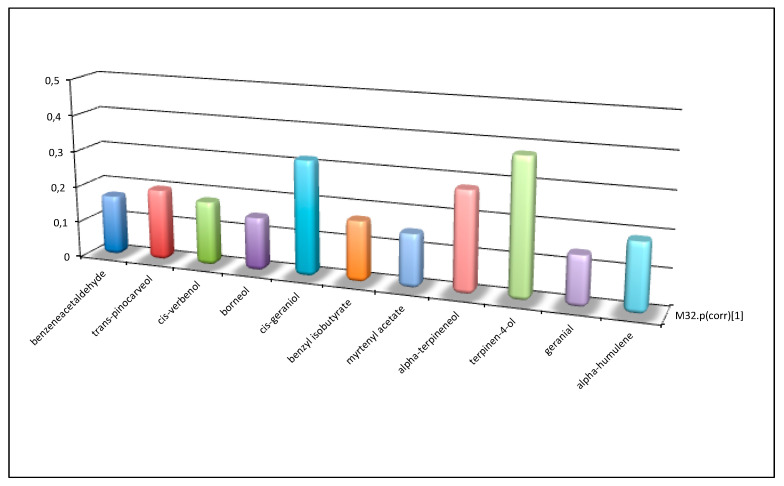
Components of essential oils influenced by ecosystem type.

**Table 1:** Sample description of the 52 genotypes growing in the Fenosu collection. A complete description of cultivars is available in the publications [2,16,17].

Locality of Origin	Code	Propagation	Commercial Name	Locality of Origin	Code	Propagation	Commercial Name
Rumanedda	RUM 6 ^w^	Agamic	Ika	Fenosu	V 12	Open pollination + agamic	Monica
Rumanedda	RUM 14 ^w^	Agamic	Grazia	Fenosu	V 15	Open pollination + agamic	Emma
Rumanedda	RUB 3 ^w^	Agamic	Angela	Fenosu	V 16	Open pollination + agamic	Lucia
Fenosu	V 3 ^w^	Open pollination + agamic	Luana	Fenosu	V 17	Open pollination + agamic	Sara M
Fenosu	V 8 ^w^	Open pollination + agamic	Caterina	Fenosu	V 19	Open pollination + agamic	Dallal
Rumanedda	RUM 3	Agamic	Simona	Fenosu	V 20	Open pollination + agamic	Surighedda
Rumanedda	RUM 4	Agamic	Giovanna	Laconi	LAC 1	Agamic	Roberta
Rumanedda	RUM 4B	Agamic	Lelia	Laconi	LAC 10	Agamic	Speranza
Rumanedda	RUM 10	Agamic	Tonina	Laconi	LAC 11	Agamic	Maria Elisa
Rumanedda	RUM 12	Agamic	Giuseppina	Laconi	LAC 31	Agamic	Sofia
Rumanedda	RUM 13	Agamic	Ilaria	Bosa	BOS 1	Agamic	Nadia
Rumanedda	RUM 15	Agamic	Erika	Bosa	BOS 2	Agamic	Marta
Rumanedda	RUM 20	Agamic	Piera	Barisardo	ORS 2	Agamic	Sara
Capoterra	CPT 3	Agamic	Maria Rita	Barisardo	ORS 3	Agamic	Michela
Capoterra	CPT 4	Agamic	Barbara	Isili	ISL 3	Agamic	Maura
Capoterra	CPT 5	Agamic	Daniela	Budoni	BUD 1	Agamic	Aurora
Capoterra	CPT 6	Agamic	Giusy	Cuglieri	CUG 11	Agamic	Maria Antonietta
Fenosu	V 1	Open pollination + agamic	Valentina	Orosei	ORO 2	Agamic	Federica
Fenosu	V 2	Open pollination + agamic	Viviana	Isili	ISL 1	Agamic	Rosella
Fenosu	V 4	Open pollination + agamic	Gian Paola	Muravera	SBD	Agamic	Veronica
Fenosu	V 5	Open pollination + agamic	Mariella	Muravera	SBD 2	Agamic	Laura
Fenosu	V 6	Open pollination + agamic	Lalla	Siniscola	SIN 2	Agamic	Carla
Fenosu	V 7	Open pollination + agamic	Greta	Monti	MON 5	Agamic	Luisa
Fenosu	V 9	Open pollination + agamic	Andrea	Telti	TEL 10	Agamic	Ana
Fenosu	V 10	Open pollination + agamic	Benedetta	Sinnai	PSF 1	Agamic	Silvia
Fenosu	V 11 ^z^	Open pollination + agamic	Enza	Sassari	SAS 1	Agamic	Antonella

^w^: samples of genotypes having white fruits; ^z^: sample of *M. communis* var. *tarantina*.

**Table 2:** Principal constituents of data reported (see Table S1).

Principal Constituent Table 1	RUM 6	RUM 14	RUB 3	V 3	V 8	RUM 3	RUM 4	RUM 4B	RUM 10	RUM 12
	(Rumanedda)	(Rumanedda)	(Rumanedda)	(Open pollination + agamic)	(Open pollination + agamic)	(Rumanedda)	(Rumanedda)	(Rumanedda)	(Rumanedda)	(Rumanedda)
α-pinene	**37.22**	**40.62**	**28.25**	**38.06**	**23.97**	**24.61**	**29.17**	**22.64**	**45.27**	**24.78**
*o*-cymene	2.06	-	0.74	-	-	1.71	0.64	3.07	1.22	0.40
limonene	**6.47**	2.31	**5.45**	**26.57**	**19.68**	2.77	3.45	**17.31**	**6.36**	**5.12**
1.8-cineole	**18.58**	**9.14**	**13.37**	**13.74**	**18.75**	**13.47**	**15.00**	**11.19**	**17.67**	**16.50**
*p*-cymenene	-	-	-	-	-	-	-	0.22	-	-
linalool	3.01	**16.48**	2.75	3.65	**13.75**	**14.53**	**10.06**	**13.10**	3.79	3.25
α-terpineol	**6.11**	4.56	**5.54**	2.49	**5.09**	**7.17**	**7.86**	**6.15**	**5.03**	**7.85**
*cis*-geraniol	1.21	-	1.77	0.57	-	4.44	2.64	1.37	0.61	1.49
linalyl acetate	-	**5.48**	-	-	-	-	-	-	-	-
myrtenyl acetate	0.05	0.06	0.06	-	-	0.96	-	-	-	0.03
geranyl acetate	4.49	3.55	**5.69**	1.28	0.69	4.96	4.31	3.71	3.18	2.01
methyleugenol	2.09	2.23	3.42	0.51	0.51	2.63	1.88	1.80	1.73	3.63
dihydroeugenyl butanoate	3.05	2.31	4.73	1.70	3.27	**5.37**	**5.07**	4.77	2.89	**8.17**

note: The data are expressed in percentages (%). Constituents that reached concentrations more than 5% are reported in bold.

**Table 3:** Principal constituents of data reported (see Table S2).

Principal Constituent Table 2	RUM 13	RUM 15	RUM 20	CPT 3	CPT 4	CPT 5	CPT 6	V1	V 2	V 4	V 5
	(Rumanedda)	(Rumanedda)	(Rumanedda)	(Capoterra)	(Capoterra)	(Capoterra)	(Capoterra)	(Open pollination + agamic)	(Open pollination + agamic)	(Open pollination + agamic)	(Open pollination + agamic)
α-pinene	**2I.86**	**19.99**	**16.99**	**33.84**	**32.50**	**23.05**	**34.14**	**24.32**	**30.67**	**32.81**	**24.17**
*o*-cymene	-	-	1.11	0.61	0.87	0.50	2.97	-	-	-	-
limonene	**17.88**	**22.72**	4.33	**7.77**	**6.30**	**19.80**	**10.66**	**19.39**	**19.47**	**5.45**	3.33
1.8-cineole	**20.60**	**8.58**	**16.72**	**20.05**	1.37	**9.44**	**14.24**	**10.09**	**24.42**	**21.21**	**16.97**
*p*-cymenene	-	-	-	-	-	-	-	-	-	-	-
linalool	3.97	**11.39**	**13.18**	3.53	4.08	**19.15**	4.68	**13.00**	2.60	3.34	**13.57**
α-terpineol	**7.87**	**5.72**	**9.13**	**6.74**	**7.95**	4.54	**5.92**	**6.43**	**8.15**	**5.80**	**8.50**
*cis*-geraniol	1.29	-	-	0.97	0.98	0.98	1.10	0.07	1.46	1.97	2.59
linalyl acetate	-	3.49	2.30	-	-	-	-	-	-	-	-
myrtenyl acetate	-	-	-	0.05	-	0.04	-	-	-	-	-
geranyl acetate	1.57	4.38	4.85	4.88	2.61	1.41	2.06	3.62	1.37	4.49	**5.42**
methyleugenol	1.21	2.11	1.94	3.43	2.12	**5.15**	0.70	2.88	1.03	3.01	2.45
dihydroeugenyl butanoate	3.05	2.31	4.73	1.70	3.27	5.37	**5.07**	4.77	2.89	8.17	

note: The data are expressed in percentages (%). Constituents that reached concentrations more than 5% are reported in bold.

**Table 4:** Principal constituents of data reported (see Table S3).

Principal Constituent Table 3	V 6	V 7	V 9	V 10	V 11	V 12	V 15	V 16	V 17	V 19	V 20
	(Open pollination + agamic)	(Open pollination + agamic)	(Open pollination + agamic)	(Open pollination + agamic)	(Open pollination + agamic)	(Open pollination + agamic)	(Open pollination + agamic)	(Open pollination + agamic)	(Open pollination + agamic)	(Open pollination + agamic)	(Open pollination + agamic)
α-pinene	**32.19**	**29.38**	**37.8**	**28.35**	**23.23**	**35.14**	**13.81**	**37.22**	**28.83**	**27.29**	**29.4**
*o*-cymene	-	-	-	-	-	-	-	-	0.49	-	0.90
limonene	4.04	**21.41**	**23.05**	3.2	4.92	**21.04**	**16.95**	**20.1**	**17.27**	**20.32**	**5.35**
1.8-cineole	**16.08**	**12.72**	**12.93**	**16.24**	**13.41**	**16.13**	**6.49**	**15.81**	**12.16**	**10.57**	**13.51**
*p*-cymenene	-	-	-	-	-	-	-	0.08	-	-	-
linalool	2.74	3.04	1.56	**13.18**	2.89	2.02	**10.32**	3.21	**10.18**	4.42	**20.14**
α-terpineol	**9.38**	4.87	4.17	**8.72**	**8.72**	**5.09**	4.06	**5.12**	**5.95**	**5.58**	**6.48**
*cis*-geraniol	0.13	0.81	0.28	-	1.25	0.32	-	-	1.25	1.29	1.09
linalyl acetate	-	-	-	3.01	-	-	1.72	-	-	-	-
myrtenyl acetate	0.06	0.03	-	-	0.08	-	**27.15**	-	-	-	0.05
geranyl acetate	**5.19**	1.08	2.49	4.78	**5.60**	2.61	2.91	0.93	3.53	1.66	1.49
methyleugenol	2.14	2.13	1.57	2.11	3.16	1.40	2.54	1.55	2.33	3.32	2.01
dihydroeugenyl butanoate	3.72	4.31	3.24	4.46	**5.16**	3.57	2.54	2.57	**6.51**	4.77	4.56

note: The data are expressed in percentages (%). Constituents that reached concentrations more than 5% are reported in bold.

**Table 5:** Principal constituents of data reported (see Table S4).

Principal Constituent Table 4	LAC 1	LAC 10	LAC 11	LAC 31	BOS 1	BOS 2	ORS 2	ORS 3	ISL 3	BUD 1
	(Laconi)	(Laconi)	(Laconi)	(Laconi)	(Bosa)	(Bosa)	(Barisardo)	(Barisardo)	(Isili)	(Budoni)
α-pinene	**35.68**	**40.94**	**31.36**	**17.35**	**32.98**	**36.50**	**39.21**	**46.51**	**16.02**	**23.89**
*o*-cymene	1.94	1.40	-	-	0.41	-	-	**6.56**	-	-
limonene	**6.46**	3.31	**8.86**	**44.99**	**12.59**	**7.83**	**8.83**	**6.55**	**5.76**	**8.05**
1.8-cineole	**11.31**	**12.74**	**22.56**	**23.74**	**16.71**	**20.86**	**21.03**	**10.83**	**13.08**	**22.94**
*p*-cymenene	-	-	**5.07**	-	-	-	-	-	-	-
linalool	3.98	0.87	-	**17.8**	3.10	4.26	1.34	2.76	**24.32**	3.07
α-terpineol	**7.32**	4.27	**7.44**	**6.38**	4.89	**6.73**	**5.06**	**5.34**	**5.32**	**8.64**
*cis*-geraniol	0.61	0.31	1.16	**8.07**	0.80	-	-	-	-	-
linalyl acetate	-	-	-	-	-	1.21	0.42	0.84	-	0.77
myrtenyl acetate	0.03	-	0.06	-	**10.36**	-	0.07	0.15	0.06	-
geranyl acetate	3.09	4.56	2.61	**5.31**	3.20	4.40	4.45	3.38	2.60	**5.96**
methyleugenol	2.38	2.86	2.53	1.41	2.30	2.48	3.74	2.56	1.82	3.34
dihydroeugenyl butanoate	**7.49**	**10.63**	2.60	0.35	2.69	2.40	4.54	2.65	**6.18**	3.68

note: The data are expressed in percentages (%). Constituents that reached concentrations more than 5% are reported in bold.

**Table 6:** Principal constituents of data reported (see Table S5).

Principal Constituent Table 5	CUG 11	ORO 2	ISL 1	SBD	SBD2	SIN 2	MON 5	TEL 10	PSF 1	SAS 1
	(Cuglieri)	(Orosei)	(Isili)	(Muravera)	(Muravera)	(Sinisacola)	(Monti)	(Telti)	(Sinnai)	(Sassari)
α-pinene	**41.61**	**26.47**	**14.46**	**32.48**	**41.08**	**29.9**	**44.58**	**33.37**	**32.74**	**14.80**
*o*-cymene	-	1.21	1.20	3.21	1.85	-	0.22	1.94	1.74	0.65
limonene	**6.96**	**5.35**	**5.61**	3.95	**8.10**	**6.67**	**8.16**	**8.05**	**6.70**	**17.52**
1.8-cineole	**16.93**	**27.12**	**11.75**	**15.79**	**23.32**	**23.06**	**15.34**	**19.82**	**22.75**	**6.41**
*p*-cymenene	-	-	-	-	-	-	-	-	-	-
linalool	2.16	3.50	**13.87**	**7.97**	2.12	4.03	2.19	4.45	1.61	**10.13**
α-terpineol	**6.10**	**7.88**	**9.36**	**8.38**	4.45	**6.41**	3.13	**5.20**	**6.21**	3.81
*cis*-geraniol	0.30	-	**5.21**	-	-	1.24	0.72	1.12	-	-
linalyl acetate	-	-	-	0.07	0.29	-	-	-	0.65	1.52
myrtenyl acetate	-	0.05	0.03	0.05	-	0.10	0.05	-	0.11	**28.13**
geranyl acetate	2.53	**5.31**	**5.83**	1.79	3.07	4.57	4.59	2.88	**5.60**	2.63
methyleugenol	1.76	3.01	2.91	2.51	0.68	2.16	2.10	1.72	2.64	2.33
dihydroeugenyl butanoate	4.42	2.78	4.30	**7.13**	0.45	2.29	1.26	4.84	**7.38**	2.31

note: The data are expressed in percentages (%). Constituents that reached concentrations more than 5% are reported in bold.

**Table 7:** Soil types of the localities under study.

	Members	Correct	Limestone	Schist	Silicon	Alluvial	
Limestone	27	100%	27	0	0	0	
Schist	5	60%	2	3	0	0	
Silicon	11	100%	0	0	11	0	
Alluvial	3	0%	3	0	0	0	
Total	46	89.13%	36	3	13	0	
Fisher’s prob.	1.30 × 10^−13^						

## Supplementary Materials

The following are available online at https://www.mdpi.com/2223-7747/9/10/1288/s1. Table S1: Leaf essential oil composition of samples of cultivars selected from Rumanedda locality and by open pollination. Table S2: Leaf essential oil composition of samples of cultivars selected from Rumanedda and Capoterra localities and by open pollination. Table S3: Leaf essential oil composition of samples of new cultivars obtained from open pollination. Table S4: Leaf essential oil composition of samples of cultivars selected from Laconi, Bosa, Rumanedda, Muravera, Isili and Budoni localities. Table S5: Leaf essential oil composition of samples of cultivars selected from Cuglieri, Orosei, Isili, Muravera, Siniscola, Monti, Telti, Sinnai and Sassari (*Myrtus communis* var. *tarantina*) localities.
