# Supplementary material for: Chemotaxonomic Evaluation by Volatolomics Analysis of Fifty-Two Genotypes of Myrtus communis L
Source: Plants (Basel). 2020 Sep 29;9(10):1288. doi: 10.3390/plants9101288 (PMC7650784; doi:10.3390/plants9101288)
Supplement: Supplementary file 1 [file plants-09-01288-s001.zip › plants-935318-supplementary.docx]

Table S1. Leaf essential oil composition of samples of cultivars selected from Rumanedda locality and by open pollination.

|  |  |  |  | **1** | **2** | **3** | **4** | **5** | **6** | **7** | **8** | **9** | **10** |  |  |
| --- | --- | --- | --- | --- | --- | --- | --- | --- | --- | --- | --- | --- | --- | --- | --- |
| **Cultivars** | | | | **RUM 6** | **RUM 14** | **RUB 3** | **V 3** | **V 8** | **RUM 3** | **RUM 4** | **RUM 4B** | **RUM 10** | **RUM 12** | ***ID** | **Literature**  **reference** |
| **Rt** | **KI lett apolar** | **KI exp apolar** | **Constituents** |  |  |  |  |  |  |  |  |  |  |  |  |
| 16.61 | 899 | 894 | isobutyl isobutyrate | 0.9±0.03 | 0.63±0.04 | 0.24±0.03 | 1.04±0.08 | 0.19±0.02 | 0.2±0.01 | 0.26±0.03 | 0.08±0.01 | 0.05±0.01 | 1.24±0.09 | MS-RI |  |
| 17.38 | 920 | 925 | β-thujene | - | - | 0.28±0.03 | - | - | - | - | - | - | - | MS-RI | [1] |
| 17.41 | 930 | 928 | α-thujene | 0.33±0.03 | 0.43±0.01 | - | 0.42±0.03 | 0.37±0.01 | 0.22±0.01 | 0.41±0.03 | 0.35±0.02 | 0.51±0.02 | 0.23±0.01 | MS-RI |  |
| 17.88 | 939 | 937 | α-pinene | 37.22±0.13 | 40.62±0.10 | 28.25±0.12 | 38.06±0.17 | 23.97±0.17 | 24.61±0.11 | 29.17±0.10 | 22.64±0.11 | 45.27±0.14 | 24.78±0.07 | Std |  |
| 18.54 | 953 | 948 | fenchene | - | - | - | - | - | - | 0.03±0.01 | 0.05±0.01 | - | - | Std |  |
| 18.63 | 955 | 954 | camphene | 0.10±0.01 | 0.17±0.02 | - | - | - | - | 0.07±0.02 | - | 0.08±0.02 | 0.06±0.01 | Std |  |
| 20.15 | 980 | 979 | β-pinene | 0.43±0.03 | - | 0.44±0.02 | 0.23±0.03 | 0.24±0.01 | 0.17±0.01 | 0.30±0.02 | 0.26±0.01 | 0.60±0.05 | 0.40±0.02 | Std |  |
| 20.80 | 992 | 991 | β-myrcene | 0.11±0.01 | 0.3±0.01 | 0.07±0.01 | - | 0.07±0.01 | 0.21±0.03 | 0.22±0.02 | 0.26±0.01 | 0.13±0.02 | 0.07±0.01 | Std |  |
| 21.40 | 1001 | 1001 | 2-methylbuthyl isobutyrate | 0.97±0.04 | 0.53±0.05 | 1.09±0.09 | 0.97±0.03 | 0.56±0.05 | 0.47±0.01 | 0.47±0.03 | 0.19±0.01 | 0.47±0.01 | 1.07±0.06 | MS-RI | [2] |
| 21.60 | 1002 | 1001 | α-phellandrene | - | 0.10±0.01 | - | 0.14±0.01 | 0.19±0.01 | - | 0.45±0.04 |  | 0.18±0.01 | 0.11±0.01 | Std |  |
| 21.93 | 1002 | 1002 | δ-2-carene | 0.40±0.01 | 0.54±0.03 | 0.22±0.01 | 0.39±0.02 | 0.47±0.03 | 0.50±0.04 | 0.55±0.05 | 0.52±0.03 | 0.53±0.02 | 0.12±0.01 | Std |  |
| 22.12 | 1013 | 1015 | 3-methyl butyl isobutyrate | 0.57±0.09 | 0.19±0.01 | 0.48±0.05 | - | 0.34±0.02 | 0.34±0.04 | 0.31±0.02 | 0.06±0.01 | 0.16±0.03 | 0.47±0.06 | MS-RI | [3] |
| 22.27 | 1017 | 1017 | α-terpinene | - | - | - | - | 0.08±0.01 | - | - | - | 0.06±0.01 | - | Std |  |
| 22.28 | 1020 | 1021 | δ-3-carene | - | 0.08±0.01 | - | - | - | - | 0.22±0.02 | - | - | 0.06±0.01 | Std |  |
| 22.66 | 1025 | 1022 | p-cymene | - | 0.76±0.05 | - | 0.89±0.05 | 1.02±0.09 | - | - | - | - | - | Std |  |
| 22.81 | 1027 | 1026 | o-cymene | 2.06±0.18 | - | 0.74±0.05 | - | - | 1.71±0.21 | 0.64±0.11 | 3.07±0.29 | 1.22±0.13 | 0.40±0.03 | Std |  |
| 22.90 | 1031 | 1029 | limonene | 6.47±0.07 | 2.31±0.04 | 5.45±0.16 | 26.57±0.17 | 19.68±0.13 | 2.77±0.05 | 3.45±0.08 | 17.31±0.11 | 6.36±0.07 | 5.12±0.05 | Std |  |
| 23.07 | 1035 | 1031 | 1,8-cineole | 18.58±0.19 | 9.14±0.16 | 13.37±0.18 | 13.74±0.09 | 18.75±0.22 | 13.47±0.21 | 15.00±0.25 | 11.19±0.14 | 17.67±0.18 | 16.50±0.19 | Std |  |
| 23.29 | 1037 | 1036 | *Cis*-β-ocimene | - | 0.27±0.03 | 0.17±0.01 | - | - | 0.24±0.02 | 0.26±0.04 | - | - | - | MS-RI |  |
| 23.77 | 1044 | 1042 | benzeneacetaldehyde | 0.09±0.01 | - | - | - | - | - | 0.02±0.01 | - | - | 0.05±0.01 | MS-RI |  |
| 23.86 | 1052 | 1050 | *Trans*-β-ocimene | - | 0.31±0.02 | 0.06±0.01 | - | 0.20±0.02 | 0.14±0.01 | 0.21±0.02 | - | 0.31±0.02 | 0.57±0.05 | MS-RI |  |
| 24.63 | 1060 | 1057 | γ-terpinene | - | 0.65±0.02 | 0.22±0.01 | 0.53±0.04 |  | 0.21±0.01 | 0.79±0.05 | - | 0.46±0.02 | 0.24±0.01 | Std |  |
| 25.35 | 1073 | 1075 | *Trans*-linalool oxide | - | - | - | - | 0.53±0.04 | - | - | - |  | 0.04±0.01 | MS-RI | [4] |
| 26.08 | 1089 | 1077 | α-terpinolene | - | 0.72±0.03 | 0.29±0.02 | 0.61±0.03 | 0.46±0.03 | 0.36±0.02 | 1.32±0.09 | - | 0.64±0.02 | 0.55±0.02 | Std |  |
| 26.13 | 1095 | 1091 | p-cymenene | - | - | - | - | - | - | - | 0.22±0.01 | - | - | MS-RI |  |
| 26.55 | 1094 | 1097 | linalool | 3.01±0.02 | 16.48±0.33 | 2.75±0.14 | 3.65±0.11 | 13.75±0.28 | 14.53±0.12 | 10.06±0.10 | 13.10±0.09 | 3.79±0.02 | 3.25±0.01 | Std |  |
| 26.77 | 1096 | 1103 | n-amyl isovalerate | 0.97±0.04 | 0.64±0.04 | 1.97±0.12 | 0.88±0.07 | 1.34±0.16 | 0.81±0.04 | 0.62±0.03 | 0.30±0.02 | 0.42±0.02 | 0.75±0.03 | MS-RI |  |
| 26.85 | 1101 | 1108 | hotrienol | - | - | - | - | - | - | - | - | - | 0.08±0.01 | MS-RI |  |
| 27.49 | 1117 | 1112 | endo-fenchol | 0.10±0.02 | - | 0.05±0.01 | - | - | - | 0.08±0.01 | 0.07±0.01 | 0.07±0.01 | 0.10±0.01 | MS-RI |  |
| 28.10 | 1130 | 1126 | α-campholenal | 0.13±0.01 | - | - | - | - | - | 0.04±0.01 | 0.11±0.02 | - | 0.04±0.01 | MS-RI |  |
| 28.55 | 1132 | 1135 | *Trans*-p-mentha-2,8-dien-1-ol | - | - | - | - | - | - | - | 0.06±0.01 | 0.15±0.01 | - | MS-RI |  |
| 28.85 | 1135 | 1135 | *Trans*-pinocarveol | 0.22±0.02 | - | 0.12±0.01 | - | - | - | - | 0.17±0.01 | - | - | MS-RI | [5] |
| 29.10 | 1143 | 1141 | *C*is-verbenol | 0.37±0.02 | - | 0.11±0.01 | - | - | - | 0.11±0.01 | 0.28±0.01 | 0.07±0.01 | 0.08±0.01 | MS-RI |  |
| 30.08 | 1165 | 1160 | pinocarvone | - | - | - | - | - | - | - | - | - | - | MS-RI |  |
| 30.20 | 1169 | 1167 | borneol | 0.20±0.02 | 0.11±0.01 | 0.13±0.02 | - | 0.08±0.01 | - | 0.18±0.01 | 0.16±0.01 | 0.13±0.02 | 0.22±0.02 | Std |  |
| 30.70 | 1177 | 1177 | terpinen-4-ol | 0.52±0.02 | 0.34±0.01 | 0.39±0.03 | 0.33±0.02 | 0.43±0.02 | 0.49±0.02 | 0.45±0.02 | 0.63±0.03 | 0.47±0.02 | 0.49±0.02 | Std |  |
| 31.15 | 1183 | 1181 | p-cymen-8-ol | 0.13±0.02 | - | 0.05±0.01 | - | 0.05±0.01 | 0.11±0.01 | 0.04±0.01 | 0.18±0.02 | 0.06±0.01 | 0.05±0.01 | MS-RI |  |
| 31.32 | 1189 | 1190 | α-terpineol | 6.11±0.06 | 4.56±0.04 | 5.54±0.12 | 2.49±0.04 | 5.09±0.11 | 7.17±0.14 | 7.86±0.12 | 6.15±0.14 | 5.03±0.09 | 7.85±0.07 | Std |  |
| 31.54 | 1190 | 1192 | methyl salicylate | - | - | - | - | - | - | - | - | - | - | MS-RI | [6] |
| 31.65 | 1196 | 1199 | myrtenol | 0.61±0.04 | 0.26±0.02 | - | 0.15±0.01 | 0.08±0.01 | 0.50±0.04 | 0.26±0.01 | 0.27±0.01 | 0.26±0.02 | 0.29±0.02 | MS-RI |  |
| 32.43 | 1205 | 1207 | verbenone | - | - | 0.08±0.01 | - | - | - | - | 0.06±0.01 | - | - | Std |  |
| 32.74 | 1217 | 1213 | *Trans*-carveol | - | - | - | - | - | - | - | 0.17±0.04 | - | 0.04±0.01 | MS-RI |  |
| 32.75 | 1120 | 1217 | endo-fenchyl acetate | - | - | - | - | - | - | - | - | - | - | MS-RI |  |
| 32.76 | 1229 | 1225 | *Cis*-carveol | 0.09±0.01 | - | 0.05±0.01 | - | 1.17±0.15 | - | - | 0.11±0.01 | - | - | MS-RI |  |
| 33.07 | 1230 | 1229 | nerol | 0.03±0.01 | 0.26±0.04 | - | - | - | 0.17±0.02 | 0.19±0.02 | - | 0.03±0.01 | 0.03±0.01 | Std |  |
| 33.15 | 1238 | 1234 | *Cis*-neral | - | - | - | - | - | - | - | 0.03±0.01 | - | - | Std |  |
| 33.21 | 1245 | 1246 | *Cis*-3-hexenyl isovalerate | - | - | - | - | - | - | 0.05±0.01 | - | - | 0.04±0.01 | MS-RI |  |
| 34.03 | 1246 | 1248 | carvone | - | - | - | - | - | - | - | 0.13±0.02 | - | - | MS-RI |  |
| 34.28 | 1253 | 1255 | *Cis*-geraniol | 1.21±0.09 | - | 1.77±0.14 | 0.57±0.03 | - | 4.44±0.32 | 2.64±0.18 | 1.37±0.14 | 0.61±0.04 | 1.49±0.06 | Std |  |
| 34.32 | 1257 | 1256 | linalyl acetate | - | 5.48±0.23 | - | - | - | - | - | - | - | - | Std |  |
| 34.32 | 1267 | 1257 | geranial | - | - | - | - | - | - | - | 0.09±0.01 | - | - | Std |  |
| 36.31 | 1269 | 1263 | benzyl isobutyrate | - | - | - | - | - | - | - | - | - | - | MS-RI |  |
| 36.33 | 1270 | 1266 | α-citral | - | - | - | - | - | - | - | - | - | - | Std |  |
| 36.39 | 1285 | 1288 | anethole | - | - | - | - | - | - | - | - | - | - | Std |  |
| 36.42 | 1289 | 1290 | bornyl acetate | - | - | - | - | - | - | - | - | - | - | Std |  |
| 36.48 | 1298 | 1298 | *Trans*-pinocarvone acetate | - | - | 0.11±0.01 | - | - | - | - | 0.08±0.01 | - | 0.05±0.01 | MS-RI |  |
| 37.28 | 1325 | 1322 | methyl geraniate | 0.11±0.01 | - | - | 0.13±0.02 | 0.10±001 | - | - | 0.07±0.01 | 0.02±0.01 | 0.11±0.02 | MS-RI |  |
| 37.50 | 1327 | 1331 | myrtenyl acetate | 0.05±0.01 | 0.06±0.01 | 0.06±0.01 | - | - | 0.96±0.04 | - | - | - | 0.03±0.01 | MS-RI |  |
| 38.14 | 1343 | 1340 | exo-2-hydroxycineole acetate | 0.21±0.01 | 0.24±0.03 | 0.27±0.02 | 0.08±0.01 | 0.07±0.01 | 0.16±0.02 | 0.14±0.02 | 0.08±0.01 | - | 0.18±0.02 | MS-RI |  |
| 38.41 | 1349 | 1351 | α-terpinyl acetate | 1.62±0.22 | 0.86±0.05 | 3.13±0.23 | - | 0.05±0.01 | 1.21±0.11 | 0.95±0.06 | 0.48±0.04 | 0.55±0.09 | - | MS-RI |  |
| 38.64 | 1357 | 1357 | benzyl-2-methyl butanoate | 0.10±0.01 | 0.08±0.01 | 0.11±0.02 | - | - | - | 0.04±0.01 | 0.10±0.01 | - | - | MS-RI |  |
| 38.71 | 1359 | 1359 | eugenol | - | - | 0.04±0.01 | - | - | - | - | - | - | 0.05±0.01 | Std |  |
| 38.79 | 1365 | 1366 | neryl acetate | 0.24±0.02 | 0.54±0.04 | 0.24±0.02 | - | - | 0.58±0.04 | 0.34±0.02 | 0.34±0.01 | 0.09±0.01 | 0.05±0.01 | Std |  |
| 39.46 | 1381 | 1379 | geranyl acetate | 4.49±0.12 | 3.55±0.10 | 5.69±0.22 | 1.28±0.10 | 0.69±0.02 | 4.96±0.21 | 4.31±0.15 | 3.71±0.09 | 3.18±0.08 | 2.01±0.04 | Std |  |
| 39.57 | 1390 | 1388 | β-cubebene | - | - | - | - | - | - | - | - | 0.07±0.01 | 0.04±0.01 | Std |  |
| 40.07 | 1391 | 1395 | β-elemene | 0.05±0.01 | - | 0.15±0.02 | - | 0.05±0.01 | - | - | 0.04±0.01 | - | 0.14±0.02 | Std |  |
| 40.25 | 1404 | 1401 | methyleugenol | 2.09±0.01 | 2.23±0.01 | 3.42±0.04 | 0.51±0.04 | 0.51±0.02 | 2.63±0.08 | 1.88±0.10 | 1.80±0.01 | 1.73±0.03 | 3.63±0.27 | MS-RI |  |
| 41.19 | 1428 | 1430 | β-caryophyllene | 0.42±0.02 | 0.31±0.02 | 1.28±0.15 | 0.41±0.02 | 0.63±0.02 | 1.38±0.02 | 1.28±0.04 | 0.44±0.02 | 1.10±0.02 | 1.80±0.04 | Std |  |
| 41.36 | 1436 | 1431 | p-Mentha-1,8-dien-7-yl acetate | - | - | - | - | - | - | - | - | - | 0.03±0.01 | MS-RI |  |
| 41.47 | 1437 | 1434 | γ-elemene | - | - | - | - | - | - | - | - | - | - | Std |  |
| 41.76 | 1440 | 1437 | α-guajene | - | - | 0,08 | - | - | - | - | - | - | - | MS-RI |  |
| 42.20 | 1441 | 1443 | aromadendrene | 0.36±0.05 | 0.73±0.04 | 0.46±0.04 | - | 0.04±0.01 | 0.22±0.02 | 0.40±0.04 | 0.14±0.01 | - | 0.06±0.01 | Std |  |
| 42.29 | 1455 | 1456 | α-humulene | 0.28±0.02 | 0.24±0.04 | 1.77±0.11 | 0.96±0.04 | 0.45±0.04 | 1.25±0.12 | 0.78±0.05 | 0.26±0.02 | 0.55±0.08 | 1.29±0.14 | Std |  |
| 42.55 | 1460 | 1458 | alloaromadendrene | - | - | 0.09±0.01 | - | - | - | - | - | - | 0.10±0.01 | MS-RI |  |
| 42.86 | 1480 | 1480 | γ-muurolene | - | - | 0.09±0.01 | - | - | - | - | - | - | 0.09±0.01 | Std |  |
| 43.02 | 1485 | 1485 | germacrene D | - | - | - | - | - | - | - | 0.06±0.01 | - | - | Std |  |
| 43.09 | 1496 | 1490 | 2-tridecanone | 0.09±0.01 | - | 0.11±0.02 | - | 0.04±0.01 | 0.07±0.01 | 0.06±0.01 | 0.12±0.02 | - | 0.16±0.02 | MS-RI |  |
| 43.22 | 1496 | 1499 | isoeugenyl methyl ether | - | - | - | - | - | - | - | - | - | 0.16±0.02 | MS-RI |  |
| 43.28 | 1497 | 1497 | β-selinene | 0.15±0.02 | - | 0.63±0.02 | - | - | - | 0.13±0.02 | - | - | - | Std |  |
| 43.52 | 1498 | 1499 | α-selinene | 0.08±0.01 | - | 0.35±0.02 | - | 0.16±0.02 | - | - | - | - | 0.43±0.03 | Std |  |
| 43.55 | 1503 | 1506 | geranyl acetate | - | - | - | - | - | - | - | - | - | - | Std |  |
| 43.57 | 1506 | 1512 | β-bisabolene | - | 0.08±0.01 | - | - | - | - | - | - | - | - | MS-RI |  |
| 43.59 | 1492 | 1512 | γ-selinene | - | - | - | - | - | - | - | - | - | 0.27±0.02 | Std |  |
| 43.61 | 1514 | 1513 | neryl butyrate | - | - | - | - | - | - | - | - | - | 0.30±0.03 | MS-RI |  |
| 43.64 | 1515 | 1515 | geranyl isobutyrate | - | - | - | 0.08±0.01 | 0.07±0.01 | - | 0.05±0.01 | 0.05±0.01 | - | - | MS-RI |  |
| 43.66 | 1515 | 1515 | *Cis*-γ-bisabolene | - | - | - | 0.1±0.01 | - | - | - | - | - | - | MS-RI |  |
| 44.06 | 1521 | 1520 | dihydroeugenyl butanoate | 3.05±0.11 | 2.31±0.15 | 4.73±0.21 | 1.70±0.09 | 3.27±0.22 | 5.37±0.21 | 5.07±0.29 | 4.77±0.17 | 2.89±0.12 | 8.17±0.33 | MS-RI | [7] |
| 44.23 | 1523 | 1520 | δ-cadinene | - | - | - | - | - | - | - | - | 0.03±0.01 | 0.16±0.02 | MS-RI |  |
| 44.84 | 1536 | 1542 | elemol | - | - | 0.11±0.02 | - | - | - | 0.18±0.01 | - | - | - | MS-RI |  |
| 44.86 | 1547 | 1550 | selina-3,7(11)-diene | - | - |  | - | - | - | 0.58±0.04 | 0.06±0.01 | - | - | MS-RI |  |
| 45.12 | 1563 | 1563 | *Trans*-nerolidol | - | - | 0.05±0.01 | - | - | - | - | 0.04±0.01 | - | 0.10±0.02 | Std |  |
| 45.72 | 1572 | 1572 | caryophyllenyl alcohol | - | - | 0.08±0.01 | - | - | - | - | - | - | 0.08±0.01 | MS-RI |  |
| 45.87 | 1578 | 1579 | spathulenol | 0.15±0.02 | - | 0.28±0.01 | - | 0.11±0.01 | 0.07±0.01 | 0.04±0.01 | 0.11±0.01 | 0.03±0.01 | 0.27±0.02 | MS-RI |  |
| 45.97 | 1580 | 1581 | *Cis*-caryophyllene oxide | 0.03±0.01 | 0.14±0.01 | - | - | 0.43±0.04 | - | 0.33±0.03 | 0.08±0.01 | 0.47±0.04 | 0.06±0.01 | MS-RI |  |
| 46.11 | 1583 | 1583 | *Trans*-caryophyllene oxide | 0.48±0.02 | - | 0.86±0.05 | 0.17±0.01 | - | 0.65±0.03 | - | 0.58±0.02 | 0.06±0.01 | 0.92±0.04 | MS-RI |  |
| 46.31 | 1585 | 1589 | globulol | - | - | 0.10±0.01 | - | - | - | - | - | - | - | MS-RI |  |
| 46.46 | 1601 | 1600 | guaiol | - | - | 0.38±0.02 | - | 0.11±0.01 | 0.13±0.01 |  | 0.16±0.01 | - | 0.10±0.01 | Std |  |
| 46.71 | 1603 | 1603 | α-dihydro(10,11)bisabolol | - | 0.35±0.02 | 0.59±0.04 | - | 0.49±0.03 | 0.19±0.01 | 0.73±0.04 |  | - | 0.44±0.02 | MS-RI |  |
| 46.78 | 1606 | 1608 | humulene epoxide II | 0.62±0.04 | - | - | 0.14±0.01 |  | 0.50±0.04 |  | 0.32±0.02 | - | 0.49±0.02 | MS-RI | [8] |
| 46.75 | 1613 | 1617 | selina-6-en-4-ol | - | - | 0.92±0.04 | - | - | - | - | - | - | - | MS-RI |  |
| 46.98 | 1631 | 1631 | dihydroeugenyl pentanoate | 1.19±0.11 | 0.98±0.08 | 3.90±0.26 | 0.80±0.04 | 2.29±0.18 | 3.68±0.33 | 3.03±0.08 | 1.99±0.18 | 0.93±0.04 | 3.17±0.26 | MS-RI | [7] |
| 47.19 | 1637 | 1632 | *Cis*-cadin-4-en-7-ol | - | - | - | - | - | - | - | - | - | - | MS-RI | [4] |
| 47.24 | 1642 | 1634 | γ-eudesmol | - | - | - | - | - | - | - | - | - | - | Std |  |
| 47.31 | 1642 | 1638 | alloaromadendrene epoxide | 0.11±0.01 | - | 0.36±0.04 | 0.06±0.01 | - | 0.17±0.02 | 0.06±0.01 | 0.06±0.01 | 0.11±0.01 | 0.20±0.02 | MS-RI |  |
| 47.37 | 1642 | 1640 | epi-α-muurolol | 0.16±0.01 | 0.12±0.01 | 0.21±0.03 | - | 0.19±0.02 | 0.25±0.02 | 0.16±0.02 | 0.09±0.01 | 0.18±0.02 | 0.32±0.04 | MS-RI |  |
| 47.41 | 1643 | 1641 | caryophylla-4(12),8(13)-dien-5β-ol | - | - | - | - | - | - | - | 0.08±0.01 | - | - | MS-RI |  |
| 47.78 | 1652 | 1655 | α-cadinol | - | 0.10±0.01 | 0.05±0.01 | - | - | - | - | 0.15±0.01 | - | 0.09±0.01 | Std |  |
| 47.81 | 1656 | 1656 | neointermedeol | - | - | 0.07±0.01 | - | 0.11±0.01 | 0.11±0.01 | 0.06±0.01 | - | 0.13±0.01 | 0.04±0.01 | MS-RI | [9] |
| 47.87 | 1660 | 1657 | α-selinen-11-en-4-ol | 0.19±0.02 | - | 0.44±0.02 | - | - | 0.11±0.01 | 0.06±0.01 | 0.07±0.01 | - | 0.41±0.04 | MS-RI |  |
| 48.18 | 1663 | 1661 | epi-globulol | 0.02±0.01 | - | 0.15±0.02 |  | 0.02±0.01 | 0.08±0.01 | 0.06±0.01 | 0.08±0.01 | 0.07±0.01 | 0.09±0.01 | MS-RI |  |
| 48.32 | 1686 | 1675 | α-bisabolol | - | - | - | - | - | - | - | - | - | - | Std |  |
| 48.54 | 1682 | 1682 | ledene oxide (I) | - | - | - | - | - | - | - | 0.03±0.01 | 0.07±0.01 | - | MS-RI | [10] |
| 48.90 | 1700 | 1713 | eudesm-7(11)-en-4-ol | 0.05±0.01 | - | 0.08±0.01 | - | - | - | 0.15±0.02 | - | 0.04±0.01 | - | MS-RI | [11] |
| 50.30 | 1760 | 1766 | benzyl benzoate | - | - | 0.04±0.01 | - | - | - | - | - | - | 0.13±0.02 | MS-RI |  |
|  |  |  |  |  |  |  |  |  |  |  |  |  |  |  |  |
|  |  |  | **Percentage of identified compounds** | **98.32** | **98.8** | **95.86** | **98.08** | **98.99** | **98.57** | **97.6** | **96.08** | **98.44** | **93.10** |  |  |
|  |  |  | **Number of identified compounds** | **53** | **42** | **65** | **31** | **45** | **45** | **58** | **63** | **50** | **73** |  |  |

Data are expressed in percentage (%). *ID = Identification methods. MS: by comparison of the mass spectrum with those of the computer mass libraries ADAMS, NIST11 and by interpretation of the mass spectra fragmentations. RI: by comparison of retention index with those reported in literature. Std: by comparison of the retention time and mass spectrum of available authentic standars. No-polar column ZB-5. Data are the mean of three replicatres ± standard deviation.

Table S2. Leaf essential oil composition of samples of cultivars selected from Rumanedda and Capoterra localities and by open pollination.

|  |  |  |  | **11** | **12** | **13** | **14** | **15** | **16** | **17** | **18** | **19** | **20** | **21** |  |  |
| --- | --- | --- | --- | --- | --- | --- | --- | --- | --- | --- | --- | --- | --- | --- | --- | --- |
| **Cultivars** | | | | **RUM 13** | **RUM 15** | **RUM 20** | **CPT 3** | **CPT 4** | **CPT 5** | **CPT 6** | **V1** | **V 2** | **V 4** | **V 5** | ***ID** | **Literature**  **reference** |
| **Rt** | **KI lett apolar** | **KI exp apolar** | **Constituents** |  |  |  |  |  |  |  |  |  |  |  |  |  |
| 16.61 | 899 | 894 | isobutyl isobutyrate | 0.45±0.04 | 0.21±0.03 | 0.22±0.02 | 1.09±0.10 | 0.2±0.01 | 0.09±0.03 | 0.24±0.06 | 0.29±0.02 | 0.56±0.04 | 0.57±0.04 | 0.19±0.01 | MS-RI |  |
| 17.38 | 920 | 925 | β-thujene | - | - | - | - | - | - | - | - | - | - | - | MS-RI | [1] |
| 17.41 | 930 | 928 | α-thujene | 0.28±0.03 | 0.41±0.02 | - | 0.29±0.01 | - | 0.27±0.04 | 0.61±0.05 | 0.35±0.01 | 0.27±0.01 | 0.32±0.02 | 0.23±0.01 | MS-RI |  |
| 17.88 | 939 | 937 | α-pinene | 29.86±0.08 | 19.99±0.07 | 16.99±0.06 | 33.84±0.10 | 32.5±0.09 | 23.05±0.09 | 34.14±0.11 | 24.32±0.24 | 30.67±0.31 | 32.81±0.21 | 24.17±0.19 | Std |  |
| 18.54 | 953 | 948 | fenchene | - | - | - | - | - | - | - | - | - | - | - | Std |  |
| 18.63 | 955 | 954 | camphene | 0.07±0.01 | 0.05±0.01 | 0.04±0.01 | 0.07±0.01 | 0.08±0.02 | 0.04±0.01 | 0.07±0.02 | 0.06±0.01 | 0.07±0.01 | - | 0.29±0.02 | Std |  |
| 20.15 | 980 | 979 | β-pinene | 0.39±0.02 | 0.23±0.01 | 0.24±0.01 | 0.41±0.02 | 0.20±0.01 | 0.19±0.01 | 0.38±0.02 | 0.19±0.01 | 0.42±0.02 | 0.32±0.03 | 0.17±0.01 | Std |  |
| 20.80 | 992 | 991 | β-myrcene | 0.15±0.01 | 0.25±0.04 | 0.2±0.01 | 0.13±0.01 | 0.05±0.01 | 0.06±0.01 | 0.10±0.01 | 0.15±0.02 | 0.15±0.01 | 0.09±0.01 | - | Std |  |
| 21.40 | 1001 | 1001 | 2-methylbuthyl isobutyrate | 0.44±0.03 | 0.20±0.01 | 0.06±0.01 | 0.65±0.09 | - | 0.09±0.02 | 0.63±0.07 | 0.57±0.04 | 0.51±0.04 | 0.94±0.07 | 0.40±0.01 | MS-RI | [2] |
| 21.60 | 1002 | 1001 | α-phellandrene | 0.23±0.01 | 0.63±0.08 | 0.28±0.02 | 0.22±0.01 | 0.06±0.01 | 0.20±0.01 | 0.25±0.03 | 0.21±0.01 | 0.22±0.02 | 0.09±0.01 | 0.13±0.02 | Std |  |
| 21.93 | 1002 | 1002 | δ-2-carene | 0.21±0.01 | 0.58±0.02 | 0.48±0.03 | 0.35±0.01 | 0.35±0.02 | 0.30±0.03 | 1.18±0.03 | 0.64±0.04 | 0.15±0.01 | 0.31±0.04 | 0.25±0.01 | Std |  |
| 22.12 | 1013 | 1015 | 3-methyl butyl isobutyrate | 0.24±0.01 | 0.16±0.01 | 0.03±0.01 | 0.37±0.05 | - | - | 0.29±0.02 | - | 0.29±0.02 | 0.62±0.08 | 0.15±0.04 | MS-RI | [3] |
| 22.27 | 1017 | 1017 | α-terpinene | - | - | - | - | - | - | - | 0.13±0.01 | 0.12±0.01 | 0.05±0.01 | 0.08±0.01 | Std |  |
| 22.28 | 1020 | 1021 | δ-3-carene | 0.12±0.01 | 0.22±0.02 | 0.12±0.01 | 0.10±0.01 | - | 0.08±0.02 | 0.12±0.01 | - | - | - | - | Std |  |
| 0 | 1025 | 1022 | p-cymene | 0.47±0.02 | 0.76±0.04 | - | - | - | - | - | 0.96±0.04 | 0.37±0.01 | 0.52±0.07 | 0.58±0.05 | Std |  |
| 22.81 | 1027 | 1026 | o-cymene | - | - | 1.11±0.11 | 0.61±0.09 | 0.87±0.08 | 0.50±0.06 | 2.97±0.33 | - | - | - | - | Std |  |
| 22.90 | 1031 | 1029 | limonene | 17.88±0.12 | 22.72±0.09 | 4.33±0.14 | 7.77±0.24 | 6.30±0.18 | 19.80±0.15 | 10.66±0.11 | 19.39±0.19 | 19.47±0.23 | 5.45±0.11 | 3.33±0.07 | Std |  |
| 23.07 | 1035 | 1031 | 1,8-cineole | 20.60±0.21 | 8.58±0.11 | 16.72±0.11 | 20.05±0.23 | 1.37±0.09 | 9.44±0.10 | 14.24±0.11 | 10.09±0.11 | 24.42±0.21 | 21.21±0.22 | 16.97±0.19 | Std |  |
| 23.29 | 1037 | 1036 | *Cis*-β-ocimene | - | 0.21±0.03 | 0.22±0.01 | 0.46±0.05 | 0.12±0.02 | 0.16±0.01 | 0.16±0.01 | 0.20±0.01 |  | 0.21±0.06 | 0.23±0.05 | MS-RI |  |
| 23.77 | 1044 | 1042 | benzeneacetaldehyde | - | - | - | - | - | - | - | - | - | - | - | MS-RI |  |
| 23.86 | 1052 | 1050 | *Trans*-β-ocimene | 0.13±0.01 | 0.18±0.01 | 0.69±0.03 | - | - | - | - | 0.12±0.01 | - | 0.07±0.01 | 0.35±0.04 | MS-RI |  |
| 24.63 | 1060 | 1057 | γ-terpinene | 0.45±0.02 | 1.04±0.09 | 0.54±0.03 | 0.55±0.04 | 0.23±0.01 | 0.49±0.02 | 0.99±0.05 | 0.86±0.05 | 0.41±0.02 | 0.41±0.02 | 0.43±0.04 | Std |  |
| 25.35 | 1073 | 1075 | *Trans*-linalool oxide | - | - | 0.06±0.01 | - | - | 0.07±0.01 | - | - | - | - | - | MS-RI | [4] |
| 26.08 | 1089 | 1077 | α-terpinolene | - | 1.42±0.14 | 0.98±0.08 | 0.82±0.07 | 0.58±0.04 | 0.67±0.02 | 1.32±0.09 | 1.07±0.09 | 0.65±0.04 | 0.47±0.02 | 0.66±0.03 | Std |  |
| 26.13 | 1095 | 1091 | p-cymenene | - | - | - | - | - | - | - | - | - | - | - | MS-RI |  |
| 26.55 | 1094 | 1097 | linalool | 3.97±0.02 | 11.39±0.21 | 13.18±0.11 | 3.53±0.04 | 4.08±0.04 | 19.15±0.18 | 4.68±0.06 | 13.00±0.10 | 2.60±0.13 | 3.34±0.22 | 13.57±0.31 | Std |  |
| 26.77 | 1096 | 1103 | n-amyl isovalerate | 0.42±0.02 | 0.27±0.02 | 0.11±0.01 | 0.62±0.08 | 0.08±0.01 | 0.10±0.01 | 1.07±0.09 | 0.80±0.04 | 0.46±0.04 | 1.40±0.11 | 0.63±0.09 | MS-RI |  |
| 26.85 | 1101 | 1108 | hotrienol | - | - | - | - | 0.13±0.01 | 0.11±0.02 | - | - | - | - | - | MS-RI |  |
| 27.49 | 1117 | 1112 | endo-fenchol | 0.08±0.01 | 0.05±0.01 | 0.06±0.01 | 0.09±0.01 | 0.10±0.02 | 0.04±0.01 | 0.07±0.01 | 0.07±0.01 | 0.09±0.01 | - | 0.07±0.01 | MS-RI |  |
| 28.10 | 1130 | 1126 | α-campholenal | - | - | - | - | - | - | - | - | - | - | - | MS-RI |  |
| 28.55 | 1132 | 1135 | *Trans*-p-mentha-2,8-dien-1-ol | - | - | - | - | - | - | - | 0.11±0.01 | - | - | - | MS-RI |  |
| 28.85 | 1135 | 1135 | *Trans*-pinocarveol | - | - | - | 0.13±0.02 | 0.07±0.01 | - | 0.09±0.01 | - | - | 0.09±0.01 | - | MS-RI | [5] |
| 29.10 | 1143 | 1141 | *Cis*-verbenol | - | - | - | - | 0.22±0.02 | - | 0.12±0.01 | - | - | - | - | MS-RI |  |
| 30.08 | 1165 | 1160 | pinocarvone | - | - | - | - | - | - | - | - | - | - | - | MS-RI |  |
| 30.20 | 1169 | 1167 | borneol | 0.16±0.01 | 0.11±0.01 | 0.18±0.02 | 0.18±0.1 | 0.19±0.03 | 0.10±0.01 | 0.11±0.01 | 0.13±0.01 | - | - | 0.16±0.02 | Std |  |
| 30.70 | 1177 | 1177 | terpinen-4-ol | 0.45±0.02 | 0.60±0.02 | 0.68±0.02 | 0.52±0.02 | 0.31±0.01 | 0.39±0.02 | 0.62±0.05 | 0.58±0.06 | 0.49±0.04 | 0.34±0.02 | 0.52±0.02 | Std |  |
| 31.15 | 1183 | 1181 | p-cymen-8-ol | - | 0.05±0.01 | 0.08±0.01 | 0.04±0.01 | 0.12±0.01 | 0.03±0.01 | 0.10±0.02 | 0.06±0.01 | - | 0.04±0.01 | 0.05±0.01 | MS-RI |  |
| 31.32 | 1189 | 1190 | α-terpineol | 7.87±0.06 | 5.72±0.04 | 9.13±0.05 | 6.74±0.04 | 7.95±0.09 | 4.54±0.04 | 5.92±0.03 | 6.43±0.04 | 8.15±0.06 | 5.80±0.38 | 8.50±0.17 | Std |  |
| 31.54 | 1190 | 1192 | methyl salicylate | - | - | - | - | - | - | - | - | - | 0.06±0.01 | - | MS-RI | [6] |
| 31.65 | 1196 | 1199 | myrtenol | 0.21±0.02 | 0.27±0.02 | 0.26±0.02 | 0.44±0.04 | 0.15±0.01 | 0.29±0.01 | 0.21±0.01 | 0.55±0.02 | 0.20±0.01 | 0.72±0.04 | 0.28±0.04 | MS-RI |  |
| 32.43 | 1205 | 1207 | verbenone | - | - | - | - | - | - | - | - | - | - | - | Std |  |
| 32.74 | 1217 | 1213 | *Trans*-carveol | - | - | - | - | - | - | - | - | - | - | - | MS-RI |  |
| 32.75 | 1120 | 1217 | endo-fenchyl acetate | - | - | - | - | - | - | - | - | - | - | - | MS-RI |  |
| 32.76 | 1229 | 1225 | *Cis*-carveol | - | - | - | 0.07±0.01 | - | - | - | 0.05±0.01 | - | - | - | MS-RI |  |
| 33.07 | 1230 | 1229 | nerol | - | 0.14±0.02 | 0.19±0.04 | 0.05±0.01 | 0.11±0.01 | 0.07±0.01 |  | 0.18±0.01 |  |  | 0.19±0.02 | Std |  |
| 33.15 | 1238 | 1234 | *Cis*-neral | - | - | - | - | - | - | - | - | - | - | - | Std |  |
| 33.21 | 1245 | 1246 | *Cis*-3-hexenyl isovalerate | - | - | 0.05±0.01 | - | - | - | - | - | - | - | - | MS-RI |  |
| 34.03 | 1246 | 1248 | carvone | - | - | - | - | - | - | - | - | - | - | - | MS-RI |  |
| 34.28 | 1253 | 1255 | *Cis*-geraniol | 1.29±0.05 | - | - | 0.97±0.08 | 0.98±0.06 | 0.98±0.06 | 1.10±0.08 | 0.07±0.01 | 1.46±0.14 | 1.97±0.24 | 2.59±0.32 | Std |  |
| 34.32 | 1257 | 1256 | linalyl acetate | - | 3.49±0.19 | 2.30±0.11 | - | - | - | - | - | - | - | - | Std |  |
| 34.32 | 1267 | 1257 | geranial | - | - | - | - | - | - | - | 1.50±0.08 | - | - | - | Std |  |
| 36.31 | 1269 | 1263 | benzyl isobutyrate | - | - | - | - | - | - | - | 0.03±0.01 | - | - | - | MS-RI |  |
| 36.33 | 1270 | 1266 | α-citral | - | - | - | - | - | - | - | - | - | - | - | Std |  |
| 36.39 | 1285 | 1288 | anethole | - | 0.02±0.01 | - | - | - | - | - | - | - | - | - | Std |  |
| 36.42 | 1289 | 1290 | bornyl acetate | - | 0.03±0.01 | - | 0.06±0.01 | - | - | - | - | - | - | - | Std |  |
| 36.48 | 1298 | 1298 | *Trans*-pinocarvone acetate | - | - | - | - | - | - | - | - | - | - | - | MS-RI |  |
| 37.28 | 1325 | 1322 | methyl geraniate | 0.12±0.01 | 0.19±0.02 | 0.18±0.02 | 0.17±0.02 | 0.17±0.02 | 0.09±0.01 | 0.11±0.02 | 0.05±0.01 | 0.11±0.01 | - | - | MS-RI |  |
| 37.50 | 1327 | 1331 | myrtenyl acetate | - | - | - | 0.05±0.01 | - | 0.04±0.01 | - | - | - | - | - | MS-RI |  |
| 38.14 | 1343 | 1340 | exo-2-hydroxycineole acetate | 0.15±0.01 | 0.10±0.01 | 0.26±0.02 | 0.27±0.02 | 0.16±0.02 | 0.04±0.01 | - | 0.13±0.01 | 0.17±0.01 | 0.23±0.02 | 0.20±0.02 | MS-RI |  |
| 38.41 | 1349 | 1351 | α-terpinyl acetate | 0.07±0.01 | 1.34±0.16 | 0.58±0.05 | 1.00±0.09 | - | - | - | 0.42±0.04 | - | 0.93±0.03 | 1.17±0.10 | MS-RI |  |
| 38.64 | 1357 | 1357 | benzyl-2-methyl butanoate | - | - | - | - | - | - | - | - | - | 0.08±0.01 | 0.11±0.01 | MS-RI |  |
| 38.71 | 1359 | 1359 | eugenol | - | 0.06±0.01 | - | - | - | 0.02±0.01 | - | - | - | - | - | Std |  |
| 38.79 | 1365 | 1366 | neryl acetate | 0.05±0.01 | 0.39±0.02 | 0.32±0.02 | 0.22±0.02 | 0.04±0.01 | 0.04±0.01 | 0.04±0.01 | 0.25±0.03 | - | - | 0.32±0.02 | Std |  |
| 39.46 | 1381 | 1379 | geranyl acetate | 1.57±0.04 | 4.38±0.11 | 4.85±0.11 | 4.88±0.14 | 2.61±0.15 | 1.41±0.08 | 2.06±0.04 | 3.62±0.05 | 1.37±0.09 | 4.49±0.22 | 5.42±0.31 | Std |  |
| 39.57 | 1390 | 1388 | β-cubebene | - | 0.04±0.01 | 0.19±0.02 | - | - | - | - | - | - | - | - | Std |  |
| 40.07 | 1391 | 1395 | β-elemene | - | - | 0.05±0.01 | 0.08±0.01 | - | 0.20±0.04 | - | - | - | - | - | Std |  |
| 40.25 | 1404 | 1401 | methyleugenol | 1.21±0.08 | 2.11±0.04 | 1.94±0.02 | 3.43±0.07 | 2.12±0.09 | 5.15±0.31 | 0.70±0.09 | 2.88±0.08 | 1.03±0.05 | 3.01±0.14 | 2.45±0.09 | MS-RI |  |
| 41.19 | 1428 | 1430 | β-caryophyllene | 0.66±0.02 | 0.66±0.05 | 2.98±0.11 | 0.98±0.07 | 3.7±0.13 | 1.44±0.08 | 0.92±0.04 | 0.41±0.04 | 0.31±0.02 | 0.86±0.02 | 1.60±0.02 | Std |  |
| 41.36 | 1436 | 1431 | p-Mentha-1,8-dien-7-yl acetate | - | - | - | - | - | - | - | - | - | - | - | MS-RI |  |
| 41.47 | 1437 | 1434 | γ-elemene | - | - | 0.08±0.01 | - | 0.07±0.01 | + | - | - | - | - | - | Std |  |
| 41.76 | 1440 | 1437 | α-guajene | - | - | 0.07±0.01 | - | 0.04±0.01 | 0.10±0.02 | - | - | - | - | - | MS-RI |  |
| 42.20 | 1441 | 1443 | aromadendrene | 0.14±0.01 | 0.16±0.02 | 0.53±0.08 | - | 0.05±0.01 | 0.08±0.01 | 0.17±0.01 | - | - | - | - | Std |  |
| 42.29 | 1455 | 1456 | α-humulene | 0.90±0.09 | 1.14±0.10 | 1.44±0.17 | 1.00±0.04 | 2.11±0.09 | 0.81±0.05 | 0.58±0.04 | 1.02±0.06 | 0.55±0.03 | 1.38±0.11 | 0.97±0.04 | Std |  |
| 42.55 | 1460 | 1458 | alloaromadendrene | - | - | - | - | - | 0.12±0.02 | - | - | - | - | - | MS-RI |  |
| 42.86 | 1480 | 1480 | γ-muurolene | - | - | 0.01±0.01 | - | - | 0.11±0.01 | - | - | - | - | - | Std |  |
| 43.02 | 1485 | 1485 | germacrene D | + | 0.49±0.02 | + | - | - | + | - | - | - | - | - | Std |  |
| 43.09 | 1496 | 1490 | 2-tridecanone | + | + | 0.13±0.02 | + | 0.09±0.01 | 0.10±0.01 | 0.10±0.02 | + | + | + | 0.05±0.01 | MS-RI |  |
| 43.22 | 1496 | 1499 | isoeugenyl methyl ether | 0.04±0.01 | 0.12±0.01 | + | + | 0.05±0.01 | 0.10±0.02 | + | 0.03±0.01 | + | 0.09±0.01 | 0.25±0.03 | MS-RI |  |
| 43.28 | 1497 | 1497 | β-selinene | 0.06±0.01 | 0.04±0.01 | 0.14±0.02 | 0.17±0.02 | 0.08±0.01 | 0.42±0.03 | + | + | 0.09±0.01 | 0.07±0.01 | + | Std |  |
| 43.52 | 1498 | 1499 | α-selinene | - | - | - | - | - | - | - | - | - | - | - | Std |  |
| 43.55 | 1503 | 1506 | geranyl acetate | - | - | - | - | - | - | - | - | - | - | - | Std |  |
| 43.57 | 1506 | 1512 | β-bisabolene | + | 0.11±0.01 | - | - | - | - | - | - | - | - | - | MS-RI |  |
| 43.59 | 1492 | 1512 | γ-selinene | - | 0.06±0.01 | 0.25±0.02 | 0.13±0.01 | 0.09±0.01 | 0.42±0.04 | - | - | - | - | - | Std |  |
| 43.61 | 1514 | 1513 | neryl butyrate | - | - | - | - | - | - | - | - | - | - | - | MS-RI |  |
| 43.64 | 1515 | 1515 | geranyl isobutyrate | 0.12±0.01 | 0.03±0.01 |  | 0.05±0.01 | 0.18±0.03 | 0.15±0.02 | 0.10±0.01 | 0.03±0.01 | 0.07±0.01 | 0.08±0.01 | 0.07±0.01 | MS-RI |  |
| 43.66 | 1515 | 1515 | *Cis*-γ-bisabolene | - | - | 0.05±0.01 | - | - | - | - | 0.09±0.01 | - | - | - | MS-RI |  |
| 44.06 | 1521 | 1520 | dihydroeugenyl butanoate | 4.17±0.15 | 4.32±0.29 | 5.22±0.26 | 2.75±0.09 | 15.21±0.13 | 3.85±0.17 | 5.90±0.34 | 3.13±0.11 | 1.68±0.07 | 3.16±0.12 | 5.88±0.24 | MS-RI | [7] |
| 44.23 | 1523 | 1520 | δ-cadinene | + | 0.04±0.01 | 0.39±0.04 | + | 0.05±0.01 | + | 0.14±0.02 | - | - | - | - | MS-RI |  |
| 44.84 | 1536 | 1542 | elemol | - | - | 0.88±0.04 | 0.11±0.01 | + | 0.13±0.02 | + | 0.07±0.01 | 0.21±0.01 | 0.20±0.02 | + | MS-RI |  |
| 44.86 | 1547 | 1550 | selina-3,7(11)-diene | 0.15±0.02 | 0.14±0.01 | 0.65±0.05 | - | 0.53±0.04 | + | 0.11±0.02 | - | - | - | 0.09±0.01 | MS-RI |  |
| 45.12 | 1563 | 1563 | *Trans*-nerolidol | - | 0.04±0.01 | 0.15±0.02 | - | 0.20±0.02 | 0.06±0.01 | 0.06±0.01 | - | - | - | - | Std |  |
| 45.72 | 1572 | 1572 | caryophyllenyl alcohol | - | + | 0.06±0.01 | - | 0.17±0.02 | - | - | - | - | - | - | MS-RI |  |
| 45.87 | 1578 | 1579 | spathulenol | - | 0.04±0.01 | 0.10±0.01 | 0.12±0.01 | 0.17±0.02 | 0.26±0.02 | 0.05±0.01 | - | - | - | - | MS-RI |  |
| 45.97 | 1580 | 1581 | *Cis*-caryophyllene oxide | 0.20±0.02 | + | 0.09±0.01 | 0.03±0.01 | 0.07±0.01 | + | 0.05±0.01 | - | - | - | - | MS-RI |  |
| 46.11 | 1583 | 1583 | *Trans*-caryophyllene oxide | + | 0.12±0.01 | 0.40±0.02 | 0.37±0.02 | 0.94±0.04 | 0.38±0.02 | 0.60±0.02 | 0.13±0.01 | 0.13±0.02 | 0.34±0.02 | 0.44±0.02 | MS-RI |  |
| 46.31 | 1585 | 1589 | globulol | - | - | 0.08±0.01 | + | 0.32±0.02 | 0.07±0.01 | + | - | - | - | - | MS-RI |  |
| 46.46 | 1601 | 1600 | guaiol | 0.15±0.02 | 0.14±0.01 | 0.25±0.03 | 0.12±0.01 | 0.79±0.05 | 0.08±0.01 | 0.16±0.01 | - | - | - | - | Std |  |
| 46.71 | 1603 | 1603 | α-dihydro(10,11)bisabolol | 0.17±0.01 | 0.16±0.01 | 0.55±0.03 | + | 0.47±0.04 | + | 0.29±0.02 | - | - | - | - | MS-RI |  |
| 46.78 | 1606 | 1608 | humulene epoxide II | 0.23±0.02 | 0.29±0.02 | + | 0.46±0.04 | 0.72±0.04 | 0.21±0.04 | 0.33±0.02 | 0.35±0.04 | 0.13±0.02 | + | 0.37±0.04 | MS-RI | [8 |
| 46.75 | 1613 | 1617 | selina-6-en-4-ol | - | - | - | - | - | 0.13±0.01 | - | - | - | - | - | MS-RI |  |
| 46.98 | 1631 | 1631 | dihydroeugenyl pentanoate | 1.41±0.17 | 0.83±0.04 | 1.83±0.22 | 0.80±0.04 | 3.10±0.19 | 0.38±0.04 | 2.47±0.01 | 1.26±0.07 | 0.75±0.04 | 2.80±0.21 | 2.83±0.19 | MS-RI | [7] |
| 47.19 | 1637 | 1632 | *Cis*-cadin-4-en-7-ol | - | - | 0.34±0.02 | - | - | - | - | - | - | - | - | MS-RI | [4] |
| 47.24 | 1642 | 1634 | γ-eudesmol | - | - | 0.11±0.01 | + | 0.19±0.02 | - | - | - | - | - | - | Std |  |
| 47.31 | 1642 | 1638 | alloaromadendrene epoxide | 0.10±0.01 | + | 0.11±0.01 | 0.18±0.02 | 0.23±0.02 | + | 0.13±0.01 | 0.15±0.01 | 0.07±0.01 | 0.29±0.02 | 0.13±0.01 | MS-RI |  |
| 47.37 | 1642 | 1640 | epi-α-muurolol | 0.13±0.01 | 0.10±0.01 | + | + | 0.59±0.09 | 0.25±0.04 | + | - | - | - | - | MS-RI |  |
| 47.41 | 1643 | 1641 | caryophylla-4(12),8(13)-dien-5β-ol | - | 0.09±0.01 | 0.34±0.06 | 0.16±0.02 | - | + | 0.15±0.02 | - | - | - | - | MS-RI |  |
| 47.78 | 1652 | 1655 | α-cadinol | - | 0.21±0.02 | 0.39±0.04 | - | - | 0.05±0.01 | + | - | - | - | - | Std |  |
| 47.81 | 1656 | 1656 | neointermedeol | - | - | - | - | 0.36±0.04 | + | 0.15±0.01 | - | - | - | - | MS-RI | [9] |
| 47.87 | 1660 | 1657 | α-selinen-11-en-4-ol | - | 0.03±0.01 | 0.15±0.02 | 0.21±0.02 | 0.22±0.02 | 0.44±0.01 | 0.07±0.01 | - | - | - | - | MS-RI |  |
| 48.18 | 1663 | 1661 | epi-globulol | - | + | 0.19±0.02 | 0.06±0.01 | 0.24±0.02 | 0.05±0.01 | 0.08±0.01 | - | - | - | - | MS-RI |  |
| 48.32 | 1686 | 1675 | α-bisabolol | - | 0.09±0.01 | - | - | 0.09±0.01 | - | - | 0.03±0.01 | - | - | - | Std |  |
| 48.54 | 1682 | 1682 | ledene oxide (I) | - | - | 0.06±0.01 | - | 0.10±0.01 | - | - | - | - | - | - | MS-RI | [10] |
| 48.90 | 1700 | 1713 | eudesm-7(11)-en-4-ol | 0.08±0.01 | 0.08±0.01 | 0.53±0.07 | - | 0.55±0.09 | 0.06±0.01 | 0.12±0.01 | - | - | - | - | MS-RI | [11] |
| 50.30 | 1760 | 1766 | benzyl benzoate | - | - | 0.03±0.01 | - | 0.10±0.02 | + | 0.05±0.01 | 0.05±0.01 |  | 0.05±0.01 | 0.13±0.02 | MS-RI |  |
|  |  |  |  |  |  |  |  |  |  |  |  |  |  |  |  |  |
|  |  |  | **Percentage of identified compounds** | **98.3** | **98.12** | **96.48** | **99.02** | **94.37** | **98.54** | **98.13** | **97.39** | **98.92** | **96.28** | **97.65** |  |  |
|  |  |  | **Number of identified compounds** | **46** | **63** | **70** | **55** | **64** | **62** | **55** | **50** | **37** | **42** | **45** |  |  |

Data are expressed in percentage (%). *ID = Identification methods. MS: by comparison of the mass spectrum with those of the computer mass libraries ADAMS, NIST11 and by interpretation of the mass spectra fragmentations. RI: by comparison of retention index with those reported in literature. Std: by comparison of the retention time and mass spectrum of available authentic standars. No-polar column ZB-5. Data are the mean of three replicatres ± standard deviation.

Table S3. Leaf essential oil composition of samples of new cultivars obtained from open pollination.

|  |  |  |  | **22** | **23** | **24** | **25** | **26** | **27** | **28** | **29** | **30** | **31** | **32** |  |  |
| --- | --- | --- | --- | --- | --- | --- | --- | --- | --- | --- | --- | --- | --- | --- | --- | --- |
| **Cultivars** | | | | **V 6** | **V 7** | **V 9** | **V 10** | **V 11** | **V 12** | **V 15** | **V 16** | **V 17** | **V 19** | **V 20** | ***ID** | **Literature**  **reference** |
| **Rt** | **KI lett apolar** | **KI exp apolar** | **Constituents** |  |  |  |  |  |  |  |  |  |  |  |  |  |
| 16.61 | 899 | 894 | isobutyl isobutyrate | 0.18±0.01 | 0.38±0.03 | 1.89±0.04 | 1.08±0.07 | 0.05±0.01 | 0.3±0.02 | 1.46±0.09 | 0.3±0.01 | 0.33±0.05 | 0.42±0.04 | 0.33±0.06 | MS-RI |  |
| 17.38 | 920 | 925 | β-thujene | - | - | - | - | - | - | - | - | - | - | - | MS-RI | [1] |
| 17.41 | 930 | 928 | α-thujene | 0.26±0.01 | 0.59±0.02 | 0.28±0.03 | 0.29±0.02 | 0.24±0.01 | 0.27±0.04 | 0.16±0.02 | 0.32±0.04 | 0.28±0.01 | 0.46±0.04 | 0.29±0.02 | MS-RI |  |
| 17.88 | 939 | 937 | α-pinene | 32.19±0.13 | 29.38±0.10 | 37.8±0.10 | 28.35±0.12 | 23.23±0.1 | 35.14±0.10 | 13.81±0.05 | 37.22±0.17 | 28.83±0.12 | 27.29±0.13 | 29.4±0.11 | Std |  |
| 18.54 | 953 | 948 | fenchene | - | - | - | - | - | - | - | - | - | 0.03±0.01 | - | Std |  |
| 18.63 | 955 | 954 | camphene | 0.07±0.01 | 0.06±0.01 | 0.08±0.02 | 0.06±0.01 | 0.04±0.01 | 0.09±0.02 | - | 0.09±0.01 | 0.07±0.01 | 0.06±0.01 | 0.05±0.01 | Std |  |
| 20.15 | 980 | 979 | β-pinene | 0.36±0.02 | 0.31±0.01 | 0.36±0.02 | 0.25±0.01 | 0.24±0.01 | 0.36±0.03 | 0.09±0.01 | 0.32±0.02 | 0.27±0.01 | 0.36±0.4 | 0.23±0.01 | Std |  |
| 20.80 | 992 | 991 | β-myrcene | 0.09±0.01 | 0.07±0.01 | 0.04±0.01 | 0.23±0.02 | 0.03±0.01 | 0.13±0.03 | 0.20±0.01 | 0.07±0.01 | 0.14±0.01 | 0.06±0.01 | - | Std |  |
| 21.40 | 1001 | 1001 | 2-methylbuthyl isobutyrate | 0.59±0.021 | 0.90±0.03 | 1.86±0.11 | 0.10±0.01 | 0.35±0.02 | 0.60±0.02 | 0.15±0.01 | 0.50±0.04 | 0.18±0.01 | 0.72±0.03 | 0.68±0.07 | MS-RI | [2] |
| 21.60 | 1002 | 1001 | α-phellandrene | 0.08±0.01 | 0.47±0.04 | 0.09±0.01 | 0.28±0.01 | 0.07±0.01 | 0.18±0.01 | 0.07±0.01 | - | 0.24±0.02 | 0.17±0.01 | 0.09±0.01 | Std |  |
| 21.93 | 1002 | 1002 | δ-2-carene | 0.25±0.02 | 0.85±0.07 | 0.05±0.01 | 0.43±0.01 | 0.38±0.02 | 0.22±0.01 | 0.21±0.01 | 0.23±0.02 | 0.30±0.02 | 0.47±0.01 | 0.45±0.01 | Std |  |
| 22.12 | 1013 | 1015 | 3-methyl butyl isobutyrate | 0.26±0.02 | 0.21±0.01 | 0.73±0.05 | 0.05±0.01 | 0.10±0.03 | 0.21±0.02 | 0.13±0.01 | 0.18±0.02 | 0.10±0.01 | 0.24±0.03 | 0.19±0.01 | MS-RI | [3 |
| 22.27 | 1017 | 1017 | α-terpinene | - | 0.17±0.02 | 0.04±0.01 | 0.14±0.02 | - | 0.08±0.01 | - | - | 0.11±0.01 | 0.06±0.01 | 0.05±0.01 | Std |  |
| 22.28 | 1020 | 1021 | δ-3-carene | - | - | - | - | - | - | - | - | - | - | - | Std |  |
| 22.66 | 1025 | 1022 | p-cymene | 0.78±0.04 | 1.38±0.10 | 0.19±0.01 | 0.68±0.01 | 1.16±0.08 | 0.51±0.04 | 0.67±0.02 | 1.38±0.12 | - | 0.98±0.09 | - | Std |  |
| 22.81 | 1027 | 1026 | o-cymene | - | - | - | - | - | - | - | - | 0.49±0.02 | - | 0.90±0.04 | Std |  |
| 22.90 | 1031 | 1029 | limonene | 4.04±0.09 | 21.41±0.15 | 23.05±0.15 | 3.20±0.09 | 4.92±0.10 | 21.04±0.14 | 16.95±0.21 | 20.10±0.18 | 17.27±0.12 | 20.32±0.12 | 5.35±0.007 | Std |  |
| 23.07 | 1035 | 1031 | 1,8-cineole | 16.08±0.15 | 12.72±0.20 | 12.93±0.18 | 16.24±0.19 | 13.41±0.19 | 16.13±0.21 | 6.49±0.15 | 15.81±0.18 | 12.16±0.09 | 10.57±0.11 | 13.51±0.15 | Std |  |
| 23.29 | 1037 | 1036 | *Cis*-β-ocimene | 0.16±0.04 | - | - | 0.27±0.02 | - | 0.22±0.02 | 0.20±0.01 | - | 0.20±0.03 | - | 0.15±0.01 | MS-RI |  |
| 23.77 | 1044 | 1042 | benzeneacetaldehyde | - | - | - | - | - | - | - | - | - | - | - | MS-RI |  |
| 23.86 | 1052 | 1050 | *Trans*-β-ocimene | 0.06±0.01 | 0.04±0.01 | 0.08±0.02 | 0.59±0.06 | 0.06±0.01 | 0.52±0.03 | 0.26±0.02 | - | 0.12±0.01 | 0.05±0.01 | - | MS-RI |  |
| 24.63 | 1060 | 1057 | γ-terpinene | 0.27±0.02 | 1.14±0.11 | 0.15±0.02 | 0.67±0.02 | 0.30±0.02 | 0.35±0.02 | 0.15±0.01 | - | 0.49±0.02 | 0.55±0.03 | 0.47±0.02 | Std |  |
| 25.35 | 1073 | 1075 | *Trans*-linalool oxide | 0.07±0.01 | - | - | - | 0.04±0.01 | - | - | - | - | 0.04±0.01 | 0.10±0.01 | MS-RI | [4] |
| 26.08 | 1089 | 1077 | α-terpinolene | 0.55±0.02 | 1.30±0.11 | 0.35±0.02 | 1.02±0.10 | 0.38±0.02 | 0.63±0.04 | 0.25±0.01 | - | 0.75±0.02 | 0.65±0.03 | 0.71±0.03 | Std |  |
| 26.13 | 1095 | 1091 | p-cymenene | - | - | - | - | - | - | - | 0.08±0.01 | - | - | - | MS-RI |  |
| 26.55 | 1094 | 1097 | linalool | 2.74±0.09 | 3.04±0.011 | 1.56±0.10 | 13.18±0.025 | 2.89±0.10 | 2.02±0.09 | 10.32±0.16 | 3.21±0.10 | 10.18±0.03 | 4.42±0.02 | 20.14±0.15 | Std |  |
| 26.77 | 1096 | 1103 | n-amyl isovalerate | 1.36±0.12 | 0.82±0.05 | 1.28±0.09 |  | 0.61±0.04 | 0.77±0.04 | - | 0.46±0.04 | 0.28±0.02 | 0.70±0.04 | 0.63±0.04 | MS-RI |  |
| 26.85 | 1101 | 1108 | hotrienol | - | - | - | 0.11±0.01 | - | - | - | - | - | - | 0.20±0.01 | MS-RI |  |
| 27.49 | 1117 | 1112 | endo-fenchol | 0.14±0.02 | 0.06±0.01 | 0.07±0.01 | 0.08±0.01 | 0.08±0.01 | 0.06±0.01 | - | 0.08±0.01 | 0.06±0.01 | 0.07±0.01 | 0.09±0.01 | MS-RI |  |
| 28.10 | 1130 | 1126 | α-campholenal | 0.07±0.01 | 0.11±0.01 | - | - | 0.12±0.02 | - | - | 0.18±0.02 | - | 0.04±0.01 | - | MS-RI |  |
| 28.55 | 1132 | 1135 | *Trans*-p-mentha-2,8-dien-1-ol | - | - | - | - | - | - | - | - | - | - | - | MS-RI |  |
| 28.85 | 1135 | 1135 | *Trans*-pinocarveol | 0.15±0.02 | - | 0.09±0.01 | - | 0.20±0.02 | - | - | 0.23±0.02 | - | 0.18±0.01 | 0.10±0.01 | MS-RI | [5] |
| 29.10 | 1143 | 1141 | *C*is-verbenol | 0.17±0.01 | 0.04±0.01 | - | - | 0.18±0.02 | - | - | 0.54±0.03 | - | 0.06±0.01 | 0.06±0.01 | MS-RI |  |
| 30.08 | 1165 | 1160 | pinocarvone | - | - | - | - | - | - | - | 0.05±0.01 | - | 0.02±0.01 | - | MS-RI |  |
| 30.20 | 1169 | 1167 | borneol | 0.24±0.02 | 0.10±0.01 | 0.12±0.01 | 0.17±0.01 | 0.23±0.02 | 0.11±0.01 | - | 0.13±0.01 | 0.13±0.02 | 0.17±0.02 | 0.16±0.01 | Std |  |
| 30.70 | 1177 | 1177 | terpinen-4-ol | 0.52±0.02 | 0.59±0.02 | 0.25±0.01 | 0.52±0.02 | 0.64±0.02 | 0.34±0.01 | 0.29±0.01 | 0.42±0.02 | 0.38±0.01 | 0.55±0.02 | 0.48±0.02 | Std |  |
| 31.15 | 1183 | 1181 | p-cymen-8-ol | 0.07±0.01 | 0.07±0.01 | - | 0.05±0.01 | 0.13±0.02 | - | - | 0.09±0.01 | - | 0.07±0.01 | 0.07±0.01 | MS-RI |  |
| 31.32 | 1189 | 1190 | α-terpineol | 9.38±0.21 | 4.87±0.09 | 4.17±0.13 | 8.72±0.15 | 8.72±0.13 | 5.09±0.04 | 4.06±0.06 | 5.12±0.05 | 5.95±0.06 | 5.58±0.08 | 6.48±0.11 | Std |  |
| 31.54 | 1190 | 1192 | methyl salicylate | 0.04±0.01 | - | - | - | - | - | - | - | - | 0.04±0.01 | - | MS-RI | [6] |
| 31.65 | 1196 | 1199 | myrtenol | 0.32±0.05 | 0.52±0.04 | 0.31±0.04 | 0.37±0.03 | 0.39±0.04 | 0.30±0.02 | 1.43±0.15 | 0.33±0.02 | 0.37±0.04 | 0.44±0.02 | 0.29±0.02 | MS-RI |  |
| 32.43 | 1205 | 1207 | verbenone | - | - | - | - | - | - | - | 0.12±0.01 | - | - | - | Std |  |
| 32.74 | 1217 | 1213 | *Trans*-carveol | - | - | - | - | - | - | - | 0.18±0.02 | - | 0.10±0.01 | - | MS-RI |  |
| 32.75 | 1120 | 1217 | endo-fenchyl acetate | - | - | - | - | - | - | - | - | - | - | - | MS-RI |  |
| 32.76 | 1229 | 1225 | *Cis*-carveol | 0.07±0.01 | 0.06±0.01 | - | - | 0.12±0.02 | - | - | - | - | - | 0.06±0.01 | MS-RI |  |
| 33.07 | 1230 | 1229 | nerol | 0.04±0.01 | 0.11±0.02 | 0.07±0.01 | 0.26±0.04 | 0.03±0.01 | 0.08±0.01 | 0.20±0.02 | - | 0.06±0.01 | 0.03±0.01 | 0.08±0.01 | Std |  |
| 33.15 | 1238 | 1234 | *Cis*-neral | - | - | - | - | - | - | - | - | - | - | - | Std |  |
| 33.21 | 1245 | 1246 | *Cis*-3-hexenyl isovalerate | - | - | - | - | 0.05±0.01 | - | - | - | - | - | - | MS-RI |  |
| 34.03 | 1246 | 1248 | carvone | - | - | - | - | - | - | - | 0.13±0.02 | - | 0.02±0.01 | - | MS-RI |  |
| 34.28 | 1253 | 1255 | *Cis*-geraniol | 0.13±0.02 | 0.81±0.04 | 0.28±0.02 | - | 1.25±0.12 | 0.32±0.04 | - | - | 1.25±0.11 | 1.29±0.013 | 1.09±0.09 | Std |  |
| 34.32 | 1257 | 1256 | linalyl acetate | - | - | - | 3.01±0.15 | - | - | 1.72±0.16 | - | - | - | - | Std |  |
| 34.32 | 1267 | 1257 | geranial | 0.89±0.09 | - | - | - | 0.06±0.01 | - | - | 0.09±0.01 | - | 0.02±0.01 | - | Std |  |
| 36.31 | 1269 | 1263 | benzyl isobutyrate | 0.05±0.01 | - | - | 0.05±0.01 | 0.02±0.01 | - | - | - | - | - | - | MS-RI |  |
| 36.33 | 1270 | 1266 | α-citral | - | - | - | - | - | - | - | - | - | - | - | Std |  |
| 36.39 | 1285 | 1288 | anethole | - | - | - | - | - | - | - | - | - | - | - | Std |  |
| 36.42 | 1289 | 1290 | bornyl acetate | - | - | - | - | - | - | - | - | - | - | - | Std |  |
| 36.48 | 1298 | 1298 | *Trans*-pinocarvone acetate | 0.12±0.02 | - | - | - | 0.08±0.01 | - | 0.39±0.04 | 0.39±0.04 | - | 0.06±0.01 | 0.06±0.01 | MS-RI |  |
| 37.28 | 1325 | 1322 | methyl geraniate | 0.08±0.01 | 0.16±0.02 | 0.07±0.01 | - | 0.22±0.02 | - | 0.10±0.01 | 0.20±0.02 | - | - | 0.17±0.02 | MS-RI |  |
| 37.50 | 1327 | 1331 | myrtenyl acetate | 0.06±0.01 | 0.03±0.01 | - | - | 0.08±0.01 | - | 27.15±0.41 | - | - | - | 0.05±0.01 | MS-RI |  |
| 38.14 | 1343 | 1340 | exo-2-hydroxycineole acetate | 0.18±0.02 | 0.10±0.01 | 0.06±0.01 | 0.19±0.01 | 0.28±0.02 | 0.10±0.01 | - | 0.06±0.01 | 0.13±0.01 | 0.07±0.01 | 0.13±0.02 | MS-RI |  |
| 38.41 | 1349 | 1351 | α-terpinyl acetate | 1.14±0.09 | 0.08±0.01 | 0.27±0.01 | 1.11±0.15 | 1.55±0.21 | 0.46±0.04 | 0.43±0.04 | - | 0.58±0.04 | 0.13±0.02 | - | MS-RI |  |
| 38.64 | 1357 | 1357 | benzyl 2-methyl butanoate | 0.17±0.02 | 0.08±0.01 | 0.06±0.01 | - | 0.14±0.02 | 0.06±0.01 | 0.11±0.02 | 0.09±0.01 | - | 0.09±0.01 | 0.08±0.01 | MS-RI |  |
| 38.71 | 1359 | 1359 | eugenol | 0.03±0.01 | - | - | - | - | - | - | - | - | 0.05±0.01 | - | Std |  |
| 38.79 | 1365 | 1366 | neryl acetate | 0.14±0.02 | 0.03±0.01 | 0.04±0.01 | 0.38±0.01 | 0.14±0.02 | 0.08±0.01 | 0.41±0.03 | 0.04±0.01 | 0.19±0.01 | 0.08±0.01 | - | Std |  |
| 39.46 | 1381 | 1379 | geranyl acetate | 5.19±0.15 | 1.08±0.05 | 2.49±0.09 | 4.78±0.18 | 5.60±0.27 | 2.61±0.12 | 2.91±0.11 | 0.93±0.04 | 3.53±0.13 | 1.66±0.04 | 1.49±0.04 | Std |  |
| 39.57 | 1390 | 1388 | β-cubebene | - | - | - | - | - | - | - | - | - | - | - | Std |  |
| 40.07 | 1391 | 1395 | β-elemene | - | - | - | - | - | - | - | - | - | 0.05±0.01 | - | Std |  |
| 40.25 | 1404 | 1401 | methyleugenol | 2.14±0.08 | 2.13±0.04 | 1.57±0.05 | 2.11±0.07 | 3.16±0.18 | 1.40±0.04 | 2.54±0.22 | 1.55±0.09 | 2.33±0.10 | 3.32±0.10 | 2.01±0.06 | MS-RI |  |
| 41.19 | 1428 | 1430 | β-caryophyllene | 2.46±0.04 | 1.04±0.04 | 0.63±0.04 | 1.91±0.01 | 1.53±0.01 | 0.67±0.04 | 0.48±0.04 | 0.65±0.10 | 0.74±0.04 | 1.51±0.04 | 0.82±0.02 | Std |  |
| 41.36 | 1436 | 1431 | p-Mentha-1,8-dien-7-yl acetate | - | - | - | - | - | - | 0.13±0.01 | - | - | - | - | MS-RI |  |
| 41.47 | 1437 | 1434 | γ-elemene | - | - | - | - | - | - | - | - | - | - | - | Std |  |
| 41.76 | 1440 | 1437 | α-guajene | - | - | - | - | 0.04±0.01 | - | - | - | - | - | - | MS-RI |  |
| 42.20 | 1441 | 1443 | aromadendrene | - | 0.14±0.02 | 0.14±0.01 | - | 0.97±0.07 | 0.14±0.01 | - | 0.23±0.04 | 0.26±0.05 | 0.76±0.04 | 0.54±0.03 | Std |  |
| 42.29 | 1455 | 1456 | α-humulene | 2.83±0.09 | 1.67±0.06 | 0.37±0.04 | 1.95±0.11 | 2.72±0.09 | 0.61±0.06 | 1.71±0.12 | 0.21±0.02 | 0.54±0.04 | 0.57±0.05 | 1.27±0.11 | Std |  |
| 42.55 | 1460 | 1458 | alloaromadendrene | - | - | - | - | - | - | - | - | - | 0.02±0.01 | - | MS-RI |  |
| 42.86 | 1480 | 1480 | γ-muurolene | - | - | - | - | - | - | - | - | - | - | - | Std |  |
| 43.02 | 1485 | 1485 | germacrene D | 0.08±0.01 | - | - | - | 0.03±0.01 | - | - | - | - | 0.14±0.02 | 0.04±0.01 | Std |  |
| 43.09 | 1496 | 1490 | 2-tridecanone | 0.08±0.01 | - | 0.05±0.01 | - | 0.09±0.01 | 0.06±0.01 | 0.06±0.01 | - | 0.04±0.01 | 0.06±0.01 |  | MS-RI |  |
| 43.22 | 1496 | 1499 | isoeugenyl methyl ether | 0.03±0.01 | 0.05±0.01 | - | - | 0.06±0.01 | - | - | - | - | 0.05±0.01 | - | MS-RI |  |
| 43.28 | 1497 | 1497 | β-selinene | - | 0.07±0.01 | - | - | 0.10±0.02 | 0.08±0.01 | - | - | - | - | - | Std |  |
| 43.52 | 1498 | 1499 | α-selinene | - | 0.04±0.01 | - | - | 0.03±0.01 | - | - | - | - | - | - | Std |  |
| 43.55 | 1503 | 1506 | geranyl acetate | - | - | - | - | - | - | - | - | - | - | - | Std |  |
| 43.57 | 1506 | 1512 | β-bisabolene | - | - | - | - | - | - | - | - | - | - | - | MS-RI |  |
| 43.59 | 1492 | 1512 | γ-selinene | - | - | - | - | - | - | - | - | - | - | - | Std |  |
| 43.61 | 1514 | 1513 | neryl butyrate | - | - | - | - | - | - | - | - | - | - | - | MS-RI |  |
| 43.64 | 1515 | 1515 | geranyl isobutyrate | 0.13±0.02 | 0.20±0.03 | - | - | 0.11±0.01 | - | - | 0.15±0.02 | - | 0.28±0.03 | 0.20±0.02 | MS-RI |  |
| 43.66 | 1515 | 1515 | *Cis*-γ-bisabolene | - | - | - | - | 0.05±0.01 | - | - | - | 0.11±0.01 | - | - | MS-RI |  |
| 44.06 | 1521 | 1520 | dihydroeugenyl butanoate | 3.72±0.14 | 4.31±0.18 | 3.24±0.21 | 4.46±0.16 | 5.16±0.30 | 3.57±0.18 | 2.54±0.17 | 2.57±0.09 | 6.51±0.24 | 4.77±0.19 | 4.56±0.19 | MS-RI | [7] |
| 44.23 | 1523 | 1520 | δ-cadinene | - | - | - | - | - | - | - | - | - | - | - | MS-RI |  |
| 44.84 | 1536 | 1542 | elemol | 0.07±0.01 | - | - | - | - | - | - | - | - | - | - | MS-RI |  |
| 44.86 | 1547 | 1550 | selina-3,7(11)-diene | - | - | - | - | 0.15±0.03 | - | - | - | 0.19±0.02 | 0.23±0.02 | 0.08±0.01 | MS-RI |  |
| 45.12 | 1563 | 1563 | *Trans*-nerolidol | 0.05±0.01 | 0.03±0.01 | - | - | 0.12±0.01 | - | - | - | - | - | 0.03±0.01 | Std |  |
| 45.72 | 1572 | 1572 | caryophyllenyl alcohol | 0.06±0.01 | - | - | - | 0.10±0.02 | - | - | - | - | 0.04±0.01 | - | MS-RI |  |
| 45.87 | 1578 | 1579 | spathulenol | - | - | - | - | 0.03±0.01 | - | - | - | - | - | - | MS-RI |  |
| 45.97 | 1580 | 1581 | *Cis*-caryophyllene oxide | 0.03±0.01 | 0.04±0.01 | 0.22±0.01 | - | 0.11±0.01 | 0.13±0.01 | - | 0.05±0.01 | 0.13±0.01 | 0.05±0.01 | 0.49±0.02 | MS-RI |  |
| 46.11 | 1583 | 1583 | *Trans*-caryophyllene oxide | 0.57±0.02 | - | - | 0.13±0.01 | 1.32±0.08 | - | 0.13±0.01 | 0.56±0.03 | - | 0.91±0.04 | - | MS-RI |  |
| 46.31 | 1585 | 1589 | globulol | - | - | - | - | 0.11±0.01 | - | - | - | - | - | - | MS-RI |  |
| 46.46 | 1601 | 1600 | guaiol | 0.33±0.02 | 0.30±0.02 | 0.07±0.01 | 0.08±0.01 | 0.90±0.04 | 0.06±0.01 | 0.10±0.01 | - | - | 0.18±0.02 | 0.30±0.02 | Std |  |
| 46.71 | 1603 | 1603 | α-dihydro(10,11)bisabolol | 0.64±0.03 | 0.35±0.02 | 0.16±0.01 | - | 0.80±0.05 | 0.32±0.02 | - | 0.15±0.01 | 0.41±0.04 | - | 0.28±0.02 | MS-RI |  |
| 46.78 | 1606 | 1608 | humulene epoxide II | 0.60±0.04 | 0.61±0.04 | 0.15±0.01 | - | 1.87±0.14 | - | 0.32±0.02 | - | - | - | 0.61±0.03 | MS-RI | [8] |
| 46.75 | 1613 | 1617 | selina-6-en-4-ol | - | - | - | 0.14±0.01 | - | - | - | 0.13±0.01 | - | 0.87±0.02 | - | MS-RI |  |
| 46.98 | 1631 | 1631 | dihydroeugenyl pentanoate | 3.06±0.23 | 2.12±0.21 | 1.28±0.09 | 0.18±0.02 | 3.55±0.30 | 2.38±0.25 | 0.15±0.01 | 1.01±0.04 | 1.43±0.19 | 2.44±0.24 | 1.88±0.11 | MS-RI | [7 |
| 47.19 | 1637 | 1632 | *Cis*-cadin-4-en-7-ol | - | - | - | - | - | - | - | - | - | - | - | MS-RI | [4] |
| 47.24 | 1642 | 1634 | γ-eudesmol | - | - | - | - | - | - | - | - | - | - | - | Std |  |
| 47.31 | 1642 | 1638 | alloaromadendrene epoxide | 0.41±0.04 | 0.27±0.02 | 0.04±0.01 | 0.11±0.01 | 0.96±0.06 | 0.03±0.01 | 0.08±0.01 | - | 0.10±0.01 | - | 0.13±0.01 | MS-RI |  |
| 47.37 | 1642 | 1640 | epi-α-muurolol | - | 0.14±0.02 | - | 0.09±0.01 | - | - | - | - | 0.05±0.01 | 0.21±0.02 | - | MS-RI |  |
| 47.41 | 1643 | 1641 | caryophylla-4(12),8(13)-dien-5β-ol | 0.23±0.02 | - | 0.08±0.01 | - | 0.24±0.02 | - | - | - | - | 0.13±0.01 | - | MS-RI |  |
| 47.78 | 1652 | 1655 | α-cadinol | - | 0.03±0.01 | - | - | - | - | - | - | 0.13±0.01 | 0.03±0.01 | - | Std |  |
| 47.81 | 1656 | 1656 | neointermedeol | - | 0.04±0.01 | - | - | 0.22±0.02 | - | - | - | - | - | 0.05±0.01 | MS-RI | [9] |
| 47.87 | 1660 | 1657 | α-selinen-11-en-4-ol | - | 0.11±0.01 | - | - | 0.18±0.02 | - | - | - | - | 0.09±0.01 | - | MS-RI |  |
| 48.18 | 1663 | 1661 | epi-globulol | - | 0.05±0.01 | - | - | 0.19±0.02 | - | - | - | - | 0.07±0.01 | 0.04±0.01 | MS-RI |  |
| 48.32 | 1686 | 1675 | α-bisabolol | - | - | - | - | - | - | - | - | 0.03±0.01 | - | - | Std |  |
| 48.54 | 1682 | 1682 | ledene oxide (I) | - | - | - | - | 0.07±0.01 | - | - | - | - | - | - | MS-RI | [10] |
| 48.90 | 1700 | 1713 | eudesm-7(11)-en-4-ol | - | 0.05±0.01 | - | - | 0.14±0.02 | - | - | - | 0.04±0.01 | 0.07±0.01 | 0.04±0.01 | MS-RI | [11] |
| 50.30 | 1760 | 1766 | benzyl benzoate | 0.21±0.04 | 0.05±0.01 | - | 0.10±0.01 | 0.13±0.01 | 0.07±0.01 | - | - | 0.08±0.01 | 0.12±0.02 | 0.07±0.01 | MS-RI |  |
|  |  |  |  |  |  |  |  |  |  |  |  |  |  |  |  |  |
|  |  |  | **Percentage of identified compounds** | **97.56** | **97.98** | **99.16** | **98.17** | **94.05** | **98.9** | **99.01** | **96.93** | **98.54** | **96.41** | **98.3** |  |  |
|  |  |  | **Number of identified compounds** | **64** | **60** | **46** | **44** | **74** | **45** | **40** | **46** | **48** | **69** | **56** |  |  |

Data are expressed in percentage (%). *ID = Identification methods. MS: by comparison of the mass spectrum with those of the computer mass libraries ADAMS, NIST11 and by interpretation of the mass spectra fragmentations. RI: by comparison of retention index with those reported in literature. Std: by comparison of the retention time and mass spectrum of available authentic standars. No-polar column ZB-5. Data are the mean of three replicatres ± standard deviation.

Table S4. Leaf essential oil composition of samples of cultivars selected from Laconi, Bosa, Rumanedda, Muravera, Isili and Budoni localities.

|  |  |  |  | **33** | **34** | **35** | **36** | **37** | **38** | **39** | **40** | **41** | **42** |  |  |
| --- | --- | --- | --- | --- | --- | --- | --- | --- | --- | --- | --- | --- | --- | --- | --- |
| **Cultivars** | | | | **LAC 1** | **LAC 10** | **LAC 11** | **LAC 31** | **BOS 1** | **BOS 2** | **ORS 2** | **ORS 3** | **ISL 3** | **BUD 1** | ***ID** | **Literature**  **reference** |
| **Rt** | **KI lett apolar** | **KI exp apolar** | **Constituents** |  |  |  |  |  |  |  |  |  |  |  |  |
| 16.61 | 899 | 894 | isobutyl isobutyrate | 0.18±0.02 | 0.07±0.01 | 0.26±0.02 | 0.41±0.04 | 0.27±0.02 | 0.12±0.01 | - | 0.13±0.03 | 1.23±0.15 | 0.12±0.02 | MS-RI |  |
| 17.38 | 920 | 925 | β-thujene | 0.4±0.01 | 0.43±0.07 | - | - | - | - | - | 0.92±0.06 | 0.22±0.02 | - | MS-RI | [1] |
| 17.41 | 930 | 928 | α-thujene | - | - | 0.55±0.02 | - | 0.22±0.01 | 0.23±0.02 | 0.26±0.01 | - | - | 0.29±0.02 | MS-RI |  |
| 17.88 | 939 | 937 | α-pinene | 35.68±0.17 | 40.94±0.14 | 31.36±0.13 | 17.35±0.09 | 32.98±0.12 | 36.5±0.11 | 39.21±0.15 | 46.51±0.16 | 16.02±0.07 | 23.89±0.11 | Std |  |
| 18.54 | 953 | 948 | fenchene | - | - | - | - | - | - | - | 0.07±0.01 | - | - | Std |  |
| 18.63 | 955 | 954 | camphene | 0.09±0.01 | 0.07±0.01 | - | - | - | 0.08±0.01 | 0.07±0.01 | 0.16±0.01 | - | 0.05±0.01 | Std |  |
| 20.15 | 980 | 979 | β-pinene | 0.34±0.01 | 0.38±0.01 | 0.36±0.01 | 0.21±0.01 | 0.34±0.02 | 0.51±0.02 | 0.40±0.01 | 0.41±0.01 | 0.15±0.01 | 0.39±0.03 | Std |  |
| 20.80 | 992 | 991 | β-myrcene | 0.10±0.01 | 0.06±0.01 | 0.09±0.02 | 0.51±0.03 | 0.10±0.01 | 0.16±0.01 | 0.10±0.01 | 0.18±0.02 | 0.06±0.01 | 0.23±0.01 | Std |  |
| 21.40 | 1001 | 1001 | 2-methylbuthyl isobutyrate | 0.44±0.04 | 0.16±0.01 | 0.17±0.01 | 0.09±0.01 | 0.24±0.01 | 0.11±0.01 | 0.14±0.01 | 0.11±0.01 | - | 0.02±0.01 | MS-RI | [2] |
| 21.60 | 1002 | 1001 | α-phellandrene | 0.21±0.01 | 0.16±0.01 | 0.66±0.09 | 0.21±0.01 | 0.10±0.02 | 0.06±0.01 | - | - | 0.11±0.01 | 0.28±0.02 | Std |  |
| 21.93 | 1002 | 1002 | δ-2-carene | 0.77±0.04 | 0.56±0.05 | 1.06±0.11 | 0.88±0.09 | 0.14±0.01 | - | - | - | 0.55±0.03 | 0.55±0.02 | Std |  |
| 22.12 | 1013 | 1015 | 3-methyl butyl isobutyrate | 0.10±0.01 | 0.06±0.01 | 0.05±0.01 | 0.06±0.01 | 0.08±0.01 | - | 0.05±0.01 | - | - | 0.11±0.01 | MS-RI | [3] |
| 22.27 | 1017 | 1017 | α-terpinene | - | - | - | - | - | - | - | - | - | 0.11±0.01 | Std |  |
| 22.28 | 1020 | 1021 | δ-3-carene | 0.09±0.01 | 0.06±0.01 | 0.24±0.05 | 0.07±0.01 | - | 0.07±0.02 | - | - | - | - | Std |  |
| 22.66 | 1025 | 1022 | p-cymene | - | - | 1.94±0.10 | 2.49±0.12 | - | 0.23±0.02 | 0.34±0.02 | - | 1.94±0.14 | 1.63±0.13 | Std |  |
| 22.81 | 1027 | 1026 | o-cymene | 1.94±0.12 | 1.40±0.11 | - | - | 0.41±0.05 | - | - | 6.56±0.25 | - | - | Std |  |
| 22.90 | 1031 | 1029 | limonene | 6.46±0.12 | 3.31±0.13 | 8.86±0.17 | 44.99±0.16 | 12.59±0.22 | 7.83±0.17 | 8.83±0.19 | 6.55±0.08 | 5.76±0.10 | 8.05±0.11 | Std |  |
| 23.07 | 1035 | 1031 | 1,8-cineole | 11.31±0.09 | 12.74±0.09 | 22.56±0.22 | 23.74±0.21 | 16.71±0.25 | 20.86±0.18 | 21.03±0.17 | 10.83±0.19 | 13.08±0.15 | 22.94±0.10 | Std |  |
| 23.29 | 1037 | 1036 | *Cis*-β-ocimene | 0.12±0.02 | - | - | 0.43±0.04 | - | 0.23±0.03 | 0.09±0.01 | - | 0.15±0.02 | 0.12±0.02 | MS-RI |  |
| 23.77 | 1044 | 1042 | benzeneacetaldehyde | - | - | - | - | - | - | - | - | - | - | MS-RI |  |
| 23.86 | 1052 | 1050 | *Trans*-β-ocimene | 0.13±0.01 | 0.08±0.01 | 0.10±0.01 | 0.57±0.04 | 0.18±0.02 | 0.19±0.01 | - | 0.06±0.01 | - | - | MS-RI |  |
| 24.63 | 1060 | 1057 | γ-terpinene | 0.64±0.02 | 0.48±0.01 | 1.43±0.10 | 0.63±0.03 | 0.31±0.02 | 0.23±0.01 | 0.09±0.01 | - | 0.23±0.01 | 0.52±0.07 | Std |  |
| 25.35 | 1073 | 1075 | *Trans*-linalool oxide | - | - | - | - | - | - | - | - | - | - | MS-RI | [4] |
| 26.08 | 1089 | 1077 | α-terpinolene | 1.11±0.07 | 0.69±0.04 | 1.81±0.13 | 0.76±0.02 | 0.45±0.03 | 0.53±0.04 | 0.26±0.01 | 0.34±0.01 | 0.54±0.02 | 0.87±0.05 | Std |  |
| 26.13 | 1095 | 1091 | p-cymenene | - | - | 5.07±0.04 | - | - | - | - | - | - | - | MS-RI |  |
| 26.55 | 1094 | 1097 | linalool | 3.98±0.04 | 0.87±0.01 | - | 17.80±0.02 | 3.10±0.04 | 4.26±0.11 | 1.34±0.05 | 2.76±0.04 | 24.32±0.21 | 3.07±0.05 | Std |  |
| 26.77 | 1096 | 1103 | n-amyl isovalerate | 0.60±0.04 | 0.26±0.02 | 0.22±0.02 | 0.17±0.02 | 0.32±0.04 | 0.18±0.01 | 0.30±0.01 | 0.08±0.01 | - | 0.29±0.01 | MS-RI |  |
| 26.85 | 1101 | 1108 | hotrienol | - | - | - | - | - | - | - | - | - | - | MS-RI |  |
| 27.49 | 1117 | 1112 | endo-fenchol | 0.13±0.02 | 0.07±0.01 | 0.09±0.01 | - | - | 0.08±0.01 | 0.06±0.01 | 0.14±0.02 | - | 0.08±0.01 | MS-RI |  |
| 28.10 | 1130 | 1126 | α-campholenal | 0.08±0.01 | - | 0.06±0.01 | - | - | - | - | 0.12±0.02 | - | - | MS-RI |  |
| 28.55 | 1132 | 1135 | *Trans*-p-mentha-2,8-dien-1-ol | - | - | - | - | - | - | - |  | - | - | MS-RI |  |
| 28.85 | 1135 | 1135 | *Trans*-pinocarveol | 0.15±0.02 | 0.10±0.01 | 0.13±0.02 | - | - | - | 0.08±0.01 | 0.21±0.02 | - | 0.11±0.01 | MS-RI | [5] |
| 29.10 | 1143 | 1141 | *C*is-verbenol | 0.08±0.01 | 0.12±0.01 | - | - | - | - | 0.11±0.01 | 0.39±0.02 | 0.09±0.01 | 0.07±0.01 | MS-RI |  |
| 30.08 | 1165 | 1160 | pinocarvone | - | - | - | - | - | - | - | - | - | - | MS-RI |  |
| 30.20 | 1169 | 1167 | borneol | 0.21±0.02 | 0.12±0.01 | 0.19±0.02 | - | 0.10±0.01 | 0.15±0.01 | 0.12±0.01 | 0.19±0.02 | 0.12±0.02 | 0.18±0.02 | Std |  |
| 30.70 | 1177 | 1177 | terpinen-4-ol | 0.73±0.04 | 0.42±0.04 | 0.84±0.09 | 0.71±0.04 | 0.34±0.02 | 0.40±0.03 | 0.32±0.02 | 0.85±0.05 | 0.56±0.04 | 0.80±0.06 | Std |  |
| 31.15 | 1183 | 1181 | p-cymen-8-ol | 0.11±0.01 | 0.07±0.01 | 0.09±0.01 | 0.10±0.02 | - | - | - | 0.23±0.01 | 0.14±0.02 | 0.04±0.01 | MS-RI |  |
| 31.32 | 1189 | 1190 | α-terpineol | 7.32±0.02 | 4.27±0.02 | 7.44±0.05 | 6.30±0.06 | 4.89±0.04 | 6.73±0.11 | 5.06±0.09 | 5.34±0.12 | 5.32±0.11 | 8.64±0.06 | Std |  |
| 31.54 | 1190 | 1192 | methyl salicylate | - | - | - | - | - | - | - | - | - | - | MS-RI | [6] |
| 31.65 | 1196 | 1199 | myrtenol | 1.19±0.12 | 0.41±0.02 | 0.55±0.04 | 0.18±0.01 | 0.62±0.04 | 0.44±0.04 | 0.32±0.02 | 0.39±0.03 | - | 0.35±0.03 | MS-RI |  |
| 32.43 | 1205 | 1207 | verbenone | - | - | - | - | - | - | - | - | - | - | Std |  |
| 32.74 | 1217 | 1213 | *Trans*-carveol | 0.07±0.01 | - | - | - | - | - | - | 0.07±0.01 | - | - | MS-RI |  |
| 32.75 | 1120 | 1217 | endo-fenchyl acetate | - | - | - | - | - | - | - | - | - | - | MS-RI |  |
| 32.76 | 1229 | 1225 | *Cis*-carveol | - | - | - | - | - | - | - | - | 0.12±0.02 | - | MS-RI |  |
| 33.07 | 1230 | 1229 | nerol | 0.09±0.01 | - | - | 0.38±0.06 | - | 0.09±0.01 | - | 0.06±0.01 | - | 0.06±0.01 | Std |  |
| 33.15 | 1238 | 1234 | *Cis*-neral | - | - | - | - | - | - | - | - | - | - | Std |  |
| 33.21 | 1245 | 1246 | *Cis*-3-hexenyl isovalerate | - | - | - | - | - | - | - | - | - | 0.07±0.01 | MS-RI |  |
| 34.03 | 1246 | 1248 | carvone | - | - | - | - | - | - | - | - | 1.08±0.18 | - | MS-RI |  |
| 34.28 | 1253 | 1255 | *Cis*-geraniol | 0.61±0.05 | 0.31±0.04 | 1.16±0.11 | 8.07±0.31 | 0.80±0.04 | - | - | - | - | - | Std |  |
| 34.32 | 1257 | 1256 | linalyl acetate | - | - | - | - | - | 1.21±0.12 | 0.42±0.06 | 0.84±0.09 | - | 0.77±0.04 | Std |  |
| 34.32 | 1267 | 1257 | geranial | - | - | - | - | - | - | - | - | - | - | Std |  |
| 36.31 | 1269 | 1263 | benzyl isobutyrate | 0.03±0.01 | 0.05±0.01 | - | - | - | - | - | - | - | - | MS-RI |  |
| 36.33 | 1270 | 1266 | α-citral | - | - | - | - | - | - | - | - | - | - | Std |  |
| 36.39 | 1285 | 1288 | anethole | - | - | - | - | - | - | - | - | - | - | Std |  |
| 36.42 | 1289 | 1290 | bornyl acetate | - | - | - | - | - | - | - | - | 0.07±0.01 | - | Std |  |
| 36.48 | 1298 | 1298 | *Trans*-pinocarvone acetate | 0.06±0.01 | 0.08±0.01 | - | - | 0.09±0.01 | - | - | 0.09±0.01 | - | - | MS-RI |  |
| 37.28 | 1325 | 1322 | methyl geraniate | 0.13±0.02 | - | 0.14±0.02 | 0.21±0.02 | - | - | - | - | 0.30±0.03 | 0.19±0.02 | MS-RI |  |
| 37.50 | 1327 | 1331 | myrtenyl acetate | 0.03±0.01 | - | 0.06±0.01 | - | 10.36±0.28 | - | 0.07±0.01 | 0.15±0.02 | 0.06±0.01 | - | MS-RI |  |
| 38.14 | 1343 | 1340 | exo-2-hydroxycineole acetate | - | 0.05±0.01 | 0.06±0.01 | 0.13±0.01 | 0.07±0.01 | - | 0.10±0.01 | 0.07±0.01 | 0.16±0.02 | 0.29±0.02 | MS-RI |  |
| 38.41 | 1349 | 1351 | α-terpinyl acetate | 0.71±0.04 | 1.86±0.18 | - | 2.46±0.10 | 0.95±0.08 | 0.68±0.04 | 2.00±0.27 | 0.51±0.05 | - | 1.48±0.11 | MS-RI |  |
| 38.64 | 1357 | 1357 | benzyl 2-methyl butanoate | 0.10±0.02 | - | 0.04±0.01 | - | - | - | - | - | - | 0.09±0.01 | MS-RI |  |
| 38.71 | 1359 | 1359 | eugenol | - | - | - | - | - | - | - | - | - | 0.19±0.02 | Std |  |
| 38.79 | 1365 | 1366 | neryl acetate | 0.20±0.02 | 0.10±0.01 | 0.04±0.01 | 0.77±0.03 | 0.12±0.01 | 0.21±0.02 | 0.10±0.01 | 0.14±0.02 | 0.09±0.01 | - | Std |  |
| 39.46 | 1381 | 1379 | geranyl acetate | 3.09±0.09 | 4.56±0.13 | 2.61±0.04 | 5.31±0.06 | 3.20±0.12 | 4.40±0.10 | 4.45±0.10 | 3.38±0.09 | 2.60±0.03 | 5.96±0.11 | Std |  |
| 39.57 | 1390 | 1388 | β-cubebene | 0.08±0.01 | 0.07±0.01 | - | - | - | - | - | - | - | 0.13±0.02 | Std |  |
| 40.07 | 1391 | 1395 | β-elemene | 0.20±0.04 | 0.17±0.04 | - | - | 0.15±0.01 | 0.32±0.08 | 0.11±0.01 | 0.05±0.01 | - | 0.06±0.01 | Std |  |
| 40.25 | 1404 | 1401 | methyleugenol | 2.38±0.11 | 2.86±0.08 | 2.53±0.08 | 1.41±0.04 | 2.30±0.10 | 2.48±0.12 | 3.74±0.10 | 2.56±0.04 | 1.82±0.04 | 3.34±0.04 | MS-RI |  |
| 41.19 | 1428 | 1430 | β-caryophyllene | 0.91±0.04 | 0.95±0.06 | 0.54±0.02 | 0.42±0.01 | 0.77±0.01 | 1.88±0.01 | 1.13±0.07 | 0.17±0.01 | 1.53±0.17 | 1.77±0.17 | Std |  |
| 41.36 | 1436 | 1431 | p-Mentha-1,8-dien-7-yl acetate | - | - | - | - | - | - | - | - | - | - | MS-RI |  |
| 41.47 | 1437 | 1434 | γ-elemene | - | - | - | - | - | - | - | - | 0.04±0.01 | - | Std |  |
| 41.76 | 1440 | 1437 | α-guajene | 0.03±0.01 | - | - | - | - | - | 0.06±0.01 | - | - | - | MS-RI |  |
| 42.20 | 1441 | 1443 | aromadendrene | 0.49±0.08 | 0.09±0.01 | 0.07±0.01 | - | 0.08±0.01 | 0.08±0.01 | 0.40±0.08 | 0.06±0.01 | - | - | Std |  |
| 42.29 | 1455 | 1456 | α-humulene | 0.52±0.04 | 0.59±0.05 | 0.31±0.02 | 1.17±0.11 | 0.81±0.05 | 0.74±0.05 | 0.29±0.02 | 0.11±0.02 | 4.96±0.21 | 1.68±0.11 | Std |  |
| 42.55 | 1460 | 1458 | alloaromadendrene | - | 0.09±0.01 | - | - | - | 0.08±0.01 | - | - | - | - | MS-RI |  |
| 42.86 | 1480 | 1480 | γ-muurolene | 0.10±0.01 | 0.10±0.01 | 0.06±0.01 | - | 0.08±0.01 | 0.11±0.01 | 0.05±0.01 | - | 0.06±0.01 | - | Std |  |
| 43.02 | 1485 | 1485 | germacrene D | - | - | - | - | - | - | - | - | - | - | Std |  |
| 43.09 | 1496 | 1490 | 2-tridecanone | 0.14±0.01 | 0.10±0.01 | 0.13±0.02 | - | - | - | 0.09±0.01 | 0.06±0.01 | 0.24±0.04 | 0.09±0.01 | MS-RI |  |
| 43.22 | 1496 | 1499 | isoeugenyl methyl ether | - | - | - | - | - | - | - | 0.02±0.01 | - | - | MS-RI |  |
| 43.28 | 1497 | 1497 | β-selinene | 0.56±0.02 | - | - | - | 0.44±0.02 | 0.38±0.01 | 0.27±0.01 | - | - | 0.08±0.01 | Std |  |
| 43.52 | 1498 | 1499 | α-selinene | - | 0.38±0.02 | 0.20±0.02 | - | - | 0.44±0.03 | - | - | - | 0.10±0.01 | Std |  |
| 43.55 | 1503 | 1506 | geranyl acetate | - | - | - | - | - | - | 0.06±0.01 | - | - | - | Std |  |
| 43.57 | 1506 | 1512 | β-bisabolene | - | - | - | - | - | - | - | - | - | - | MS-RI |  |
| 43.59 | 1492 | 1512 | γ-selinene | 0.44±0.05 | 0.30±0.03 | 0.16±0.01 | - | 0.35±0.02 | - | 0.20±0.02 | - | - | - | Std |  |
| 43.61 | 1514 | 1513 | neryl butyrate | - | - | - | - | - | - | - | - | - | - | MS-RI |  |
| 43.64 | 1515 | 1515 | geranyl isobutyrate | - | 0.02±0.01 | 0.10±0.02 | - | - | - | - | - | 0.35±0.04 | 0.07±0.01 | MS-RI |  |
| 43.66 | 1515 | 1515 | *Cis*-γ-bisabolene | - | - | - | - | - | - | - | - | - | - | MS-RI |  |
| 44.06 | 1521 | 1520 | dihydroeugenyl butanoate | 7.49±0.22 | 10.63±0.13 | 2.60±0.09 | 0.35±0.04 | 2.69±0.11 | 2.40±0.12 | 4.54±0.21 | 2.65±0.11 | 6.18±0.17 | 3.68±0.08 | MS-RI | [7] |
| 44.23 | 1523 | 1520 | δ-cadinene | 0.06±0.01 | 0.06±0.01 | - | - | - | - | - | - | 0.17±0.02 | 0.06±0.01 | MS-RI |  |
| 44.84 | 1536 | 1542 | elemol | 0.05±0.01 | 0.08±0.01 | - | - | - | - | 0.06±0.01 | 0.11±0.02 | 0.07±0.01 | 0.12±0.02 | MS-RI |  |
| 44.86 | 1547 | 1550 | selina-3,7(11)-diene | - | - | - | - | - | 0.07±0.01 | - | - | - | - | MS-RI |  |
| 45.12 | 1563 | 1563 | *Trans*-nerolidol | - | 0.03±0.01 | - | - | - | - | - | 0.04±0.01 | 0.08±0.01 | 0.03±0.01 | Std |  |
| 45.72 | 1572 | 1572 | caryophyllenyl alcohol | - | - | - | - | - | - | - | - | - | - | MS-RI |  |
| 45.87 | 1578 | 1579 | spathulenol | 0.12±0.01 | 0.42±0.04 | 0.24±0.02 | - | 0.24±0.02 | 0.29±0.02 | 0.16±0.02 | 0.11±0.01 | - | 0.06±0.01 | MS-RI |  |
| 45.97 | 1580 | 1581 | *Cis*-caryophyllene oxide | - | 0.53±0.04 | 0.25±0.02 | 0.17±0.01 | - | - | - | - | 0.06±0.01 | - | MS-RI |  |
| 46.11 | 1583 | 1583 | *Trans*-caryophyllene oxide | 0.19±0.01 | 0.09±0.01 | - | - | 0.26±0.01 | 0.44±0.04 | 0.25±0.02 | 0.17±0.01 | 0.56±0.04 | 0.45±0.02 | MS-RI |  |
| 46.31 | 1585 | 1589 | globulol | - | 0.08±0.01 | 0.05±0.01 | - | - | 0.11±0.01 | - | - | 0.11±0.02 | 0.06±0.01 | MS-RI |  |
| 46.46 | 1601 | 1600 | guaiol | 0.23±0.01 | 0.22±0.01 | - | - | - | 0.07±0.01 | - | 0.14±0.01 | - | 0.26±0.03 | Std |  |
| 46.71 | 1603 | 1603 | α-dihydro(10,11)bisabolol | 0.06±0.01 | 0.29±0.02 | - | - | - | - | - | - | - | - | MS-RI |  |
| 46.78 | 1606 | 1608 | humulene epoxide II | 0.42±0.04 | - | 0.16±0.01 | 0.30±0.01 | 0.24±0.02 | 0.15±0.02 | 0.31±0.03 | 0.40±0.04 | 1.42±0.17 | 0.64±0.04 | MS-RI | [8] |
| 46.75 | 1613 | 1617 | selina-6-en-4-ol | 0.17±0.02 | 0.23±0.02 | 0.10±0.01 | - | 0.11±0.01 | - | 0.07±0.01 | - | - | - | MS-RI |  |
| 46.98 | 1631 | 1631 | dihydroeugenyl pentanoate | 1.83±0.19 | 2.49±0.01 | 0.45±0.04 | 0.10±0.01 | 0.62±0.07 | 0.60±0.05 | 1.44±0.11 | 0.49±0.04 | 0.29±0.02 | 1.44±0.11 | MS-RI | [7] |
| 47.19 | 1637 | 1632 | *Cis*-cadin-4-en-7-ol | - | - | - | - | - | - | - | - | - | - | MS-RI | [4] |
| 47.24 | 1642 | 1634 | γ-eudesmol | - | - | - | - | - | - | - | - | 0.09±0.01 | 0.27±0.02 | Std |  |
| 47.31 | 1642 | 1638 | alloaromadendrene epoxide | 0.08±0.01 | 0.07±0.01 | - | 0.04±0.01 | - | 0.06±0.01 | - | - | 0.50±0.04 | - | MS-RI |  |
| 47.37 | 1642 | 1640 | epi-α-muurolol | 0.21±0.04 | 0.23±0.04 | 0.23±0.03 | - | 0.14±0.02 | 0.21±0.02 | 0.13±0.01 | 0.16±0.01 | 0.17±0.01 | - | MS-RI |  |
| 47.41 | 1643 | 1641 | caryophylla-4(12),8(13)-dien-5β-ol | - | - | - | - | - | - | - | - | 0.21±0.03 | 0.28±0.02 | MS-RI |  |
| 47.78 | 1652 | 1655 | α-cadinol | 0.03±0.01 | 0.05±0.01 | - | - | - | - | - | - | 0.68±0.04 | 0.31±0.02 | Std |  |
| 47.81 | 1656 | 1656 | neointermedeol | - | - | - | - | - | 0.09±0.01 | - | 0.04±0.01 | - | - | MS-RI | [9] |
| 47.87 | 1660 | 1657 | α-selinen-11-en-4-ol | 0.35±0.04 | 0.39±0.04 | 0.28±0.02 | - | 0.33±0.05 | 0.74±0.09 | 0.18±0.01 | - | 0.08±0.01 | 0.19±0.02 | MS-RI |  |
| 48.18 | 1663 | 1661 | epi-globulol | 0.04±0.01 | 0.04±0.01 | - | - | - | 0.06±0.01 | - | - | 0.01±0.01 | 0.08±0.01 | MS-RI |  |
| 48.32 | 1686 | 1675 | α-bisabolol | - | - | - | - | - | - | - | - | - | 0.03±0.01 | Std |  |
| 48.54 | 1682 | 1682 | ledene oxide (I) | - | - | - | - | - | - | - | - | - | - | MS-RI | [10] |
| 48.90 | 1700 | 1713 | eudesm-7(11)-en-4-ol | - | - | 0.06±0.01 | - | - | 0.14±0.02 | - | 0.05±0.01 | 0.14±0.02 | 0.12±0.01 | MS-RI | [11] |
| 50.30 | 1760 | 1766 | benzyl benzoate | 0.10±0.01 | 0.18±0.01 | - | - | - | - | - | - | - | 0.05±0.01 | MS-RI |  |
|  |  |  |  |  |  |  |  |  |  |  |  |  |  |  |  |
|  |  |  | **Percentage of identified compounds** | **97.09** | **97.2** | **98.81** | **99.95** | **99.69** | **98.71** | **99.26** | **96.23** | **94.89** | **98.35** |  |  |
|  |  |  | **Number of identified compounds** | **67** | **64** | **52** | **37** | **45** | **51** | **48** | **51** | **51** | **63** |  |  |

Data are expressed in percentage (%). *ID = Identification methods. MS: by comparison of the mass spectrum with those of the computer mass libraries ADAMS, NIST11 and by interpretation of the mass spectra fragmentations. RI: by comparison of retention index with those reported in literature. Std: by comparison of the retention time and mass spectrum of available authentic standars. No-polar column ZB-5. Data are the mean of three replicatres ± standard deviation.

Table S5. Leaf essential oil composition of samples of cultivars selected from Cuglieri, Orosei, Isili, Muravera, Siniscola, Monti, Telti, Sinnai and Sassari (*Myrtus communis* var. *tarantina*) localities.

|  | | | | **43** | **44** | **45** | **46** | **47** | **48** | **49** | **50** | **51** | **52** |  |  |
| --- | --- | --- | --- | --- | --- | --- | --- | --- | --- | --- | --- | --- | --- | --- | --- |
| **Cultivars** | | | | **CUG 11** | **ORO 2** | **ISL 1** | **SBD** | **SBD2** | **SIN 2** | **MON 5** | **TEL 10** | **PSF 1** | **SAS 1** | ***ID** | **Literature**  **reference** |
| **Rt** | **KI lett apolar** | **KI exp apolar** | **Constituents** |  |  |  |  |  |  |  |  |  |  |  |  |
| 16.61 | 899 | 894 | isobutyl isobutyrate | 0.36±0.01 | 0.21±0.02 | 0.08±0.01 | 0.58±0.04 | 0.25±0.03 | 0.25±0.01 | 0.54±0.03 | 1.29±0.19 | 0.15±0.01 | 1.50±0.12 | MS-RI |  |
| 17.38 | 920 | 925 | β-thujene | 0.56±0.04 | 0.23±0.01 | - | 0.48±0.05 | - | 0.56±0.02 | - | - | - | - | MS-RI | [1] |
| 17.41 | 930 | 928 | α-thujene | - | - | 0.22±0.01 | - | 0.78±0.03 | - | 0.3±0.02 | 0.37±0.02 | 0.38±0.03 | 0.16±0.02 | MS-RI |  |
| 17.88 | 939 | 937 | α-pinene | 41.61±0.14 | 26.47±0.09 | 14.46±0.05 | 32.48±0.11 | 41.08±0.14 | 29.9±0.09 | 44.58±0.17 | 33.37±0.16 | 32.74±0.13 | 14.8±0.04 | Std |  |
| 18.54 | 953 | 948 | fenchene | - | - | 0.02±0.01 | 0.04±0.01 | 0.05±0.01 | - | - | - | - | - | Std |  |
| 18.63 | 955 | 954 | camphene | 0.10±0.01 | - | 0.04±0.01 | 0.11±0.02 | 0.11±0.02 | 0.05±0.01 | 0.08±0.02 | - | 0.07±0.01 | - | Std |  |
| 20.15 | 980 | 979 | β-pinene | 0.46±0.03 | 0.39±0.02 | 0.19±0.01 | 0.32±0.01 | 0.77±0.01 | 0.50±0.04 | 0.72±0.03 | 0.40±0.02 | 0.46±0.01 | 0.09±0.01 | Std |  |
| 20.80 | 992 | 991 | β-myrcene | 0.14±0.01 | 0.13±0.01 | 0.52±0.05 | 0.07±0.01 | 0.14±0.01 | 0.27±0.01 | 0.21±0.02 | 0.07±0.01 | 0.12±0.01 | 0.19±0.01 | Std |  |
| 21.40 | 1001 | 1001 | 2-methylbuthyl isobutyrate | 0.47±0.02 | 0.43±0.05 | 0.05±0.01 | 0.05±0.01 | 0.48±0.04 | 0.15±0.01 | 0.76±0.07 | 0.90±0.08 | 0.18±0.01 | 0.12±0.01 | MS-RI | [2] |
| 21.60 | 1002 | 1001 | α-phellandrene | 0.53±0.09 | 0.06±0.01 | 0.30±0.01 | 0.05±0.01 | 0.62±0.04 | 0.90±0.08 | - | - | 0.12±0.01 | - | Std |  |
| 21.93 | 1002 | 1002 | δ-2-carene | 0.80±0.05 | 0.25±0.01 | 0.52±0.01 | 0.87±0.07 | 1.36±0.12 | 0.80±0.03 | - | 0.41±0.04 | 0.44±0.03 | 0.22±0.02 | Std |  |
| 22.12 | 1013 | 1015 | 3-methyl butyl isobutyrate | 0.14±0.01 | 0.15±0.01 | - | - | 0.11±0.02 | 0.05±0.01 | 0.31±0.03 | 0.34±0.04 | - | 0.13±0.01 | MS-RI | [3] |
| 22.27 | 1017 | 1017 | α-terpinene | 0.20±0.03 | - | - | - | 0.33±0.03 | - | - | - | - | - | Std |  |
| 22.28 | 1020 | 1021 | δ-3-carene | - | - | 0.15±0.01 | - | - | 0.29±0.07 | - | - | - | - | Std |  |
| 22.66 | 1025 | 1022 | p-cymene | 1.16±0.11 | - | - | - | - | 1.21±0.09 | - | - | - | - | Std |  |
| 22.81 | 1027 | 1026 | o-cymene | - | 1.21±0.09 | 1.20±0.17 | 3.21±0.19 | 1.85±0.19 | - | 0.22±0.01 | 1.94±0.17 | 1.74±0.21 | 0.65±0.09 | Std |  |
| 22.90 | 1031 | 1029 | limonene | 6.96±0.11 | 5.35±0.09 | 5.61±0.18 | 3.95±0.09 | 8.10±0.12 | 6.67±0.21 | 8.16±0.18 | 8.05±0.19 | 6.7±0.08 | 17.52±0.25 | Std |  |
| 23.07 | 1035 | 1031 | 1,8-cineole | 16.93±0.19 | 27.12±0.24 | 11.75±0.09 | 15.79±0.20 | 23.32±0.28 | 23.06±0.26 | 15.34±0.19 | 19.82±0.18 | 22.75±0.19 | 6.41±0.11 | Std |  |
| 23.29 | 1037 | 1036 | *Cis*-β-ocimene | 0.16±0.01 | 0.13±0.01 | 0.39±0.04 | - | 0.33±0.03 | 1.06±0.05 | 0.24±0.02 | - | 0.18±0.02 | 0.21±0.02 | MS-RI |  |
| 23.77 | 1044 | 1042 | benzeneacetaldehyde | - | - | 0.03±0.01 | 0.08±0.01 | - | - | - | - | - | - | MS-RI |  |
| 23.86 | 1052 | 1050 | *Trans*-β-ocimene | - | - | 0.72±0.06 | 0.06±0.01 | - | - | - | - | - | 0.24±0.02 | MS-RI |  |
| 24.63 | 1060 | 1057 | γ-terpinene | 1.02±0.09 | 0.09±0.01 | 0.77±0.06 | 0.07±0.01 | 1.91±0.12 | 1.18±0.10 | 0.12±0.01 | - | 0.19±0.01 | 0.14±0.01 | Std |  |
| 25.35 | 1073 | 1075 | *Trans*-linalool oxide | - | - | 0.05±0.01 | 0.07±0.01 | - | - | - | - | - | - | MS-RI | [4] |
| 26.08 | 1089 | 1077 | α-terpinolene | 1.40±0.11 | 0.27±0.02 | 1.29±0.09 | 0.41±0.02 | 1.98±0.10 | 1.60±0.12 | 0.32±0.02 | - | 0.44±0.02 | 0.25±0.01 | Std |  |
| 26.13 | 1095 | 1091 | p-cymenene | - | - | - | - | - | - | - | - | - | - | MS-RI |  |
| 26.55 | 1094 | 1097 | linalool | 2.16±0.03 | 3.50±0.04 | 13.87±0.26 | 7.97±0.08 | 2.12±0.02 | 4.03±0.07 | 2.19±0.04 | 4.45±0.04 | 1.61±0.04 | 10.13±0.15 | Std |  |
| 26.77 | 1096 | 1103 | n-amyl isovalerate | 0.38±0.03 | 0.52±0.05 | 0.03±0.01 | - | 0.46±0.02 | 0.11±0.01 | 1.21±0.09 | 0.65±0.04 | 0.11±0.01 | - | MS-RI |  |
| 26.85 | 1101 | 1108 | hotrienol | - | - | 0.15±0.02 | - | - | 0.05±0.01 | - | - | - | - | MS-RI |  |
| 27.49 | 1117 | 1112 | endo-fenchol | 0.08±0.01 | 0.10±0.01 | 0.08±0.01 | 0.14±0.02 | 0.08±0.01 | 0.05±0.01 | - | 0.06±0.01 | 0.06±0.01 | - | MS-RI |  |
| 28.10 | 1130 | 1126 | α-campholenal | - | 0.09±0.01 | - | 0.11±0.02 | - | - | - | 0.13±0.02 | - | - | MS-RI |  |
| 28.55 | 1132 | 1135 | *Trans*-p-mentha-2,8-dien-1-ol | - | - | - | - | - | - | - | - | - | - | MS-RI |  |
| 28.85 | 1135 | 1135 | *Trans*-pinocarveol | - | 0.21±0.01 | - | 0.23±0.02 | 0.06±0.01 | 0.07±0.01 | - | 0.14±0.01 | - | - | MS-RI | [5] |
| 29.10 | 1143 | 1141 | *C*is-verbenol | - | 0.21±0.04 | 0.06±0.01 | 0.27±0.02 | - | - | - | 0.28±0.02 | 0.10±0.01 | - | MS-RI |  |
| 30.08 | 1165 | 1160 | pinocarvone | - | - | - | - | - | - | - | - | - | - | MS-RI |  |
| 30.20 | 1169 | 1167 | borneol | 0.15±0.02 | 0.18±0.01 | 0.16±0.02 | 0.30±0.04 | 0.09±0.01 | 0.14±0.01 | 0.10±0.03 | 0.14±0.01 | 0.12±0.02 | - | Std |  |
| 30.70 | 1177 | 1177 | terpinen-4-ol | 0.57±0.02 | 0.69±0.02 | 0.66±0.04 | 0.82±0.04 | 0.79±0.04 | 0.66±0.04 | 0.30±0.03 | 0.46±0.03 | 0.61±0.02 | 0.26±0.02 | Std |  |
| 31.15 | 1183 | 1181 | p-cymen-8-ol | - | 0.12±0.02 | 0.03±0.01 | 0.08±0.01 | - | 0.05±0.01 | - | - | - | - | MS-RI |  |
| 31.32 | 1189 | 1190 | α-terpineol | 6.10±0.17 | 7.88±0.09 | 9.36±0.08 | 8.38±0.21 | 4.45±0.10 | 6.41±0.04 | 3.13±0.05 | 5.20±0.08 | 6.21±0.15 | 3.81±0.08 | Std |  |
| 31.54 | 1190 | 1192 | methyl salicylate | - | - | - | - | - | - | - | - | - | - | MS-RI | [6] |
| 31.65 | 1196 | 1199 | myrtenol | - | - | 0.69±0.04 | 0.04±0.01 | 0.35±0.04 | - | - | 0.28±0.02 | 0.25±0.02 | 1.33±0.22 | MS-RI |  |
| 32.43 | 1205 | 1207 | verbenone | - | 0.08±0.01 | - | 0.06±0.01 | - | - | - | - | - | - | Std |  |
| 32.74 | 1217 | 1213 | *Trans*-carveol | - | 0.04±0.01 | 0.02±0.01 | - | - | 0.06±0.01 | - | 0.08±0.01 | - | - | MS-RI |  |
| 32.75 | 1120 | 1217 | endo-fenchyl acetate | - | - | 0.02±0.01 | - | - | - | - | - | - | - | MS-RI |  |
| 32.76 | 1229 | 1225 | *Cis-*carveol | - | 0.06±0.01 |  | 0.07±0.01 | - | - | - | - | - | - | MS-RI |  |
| 33.07 | 1230 | 1229 | nerol | - | - | 0.72±0.06 | - | - | - | - | - | - | 0.18±0.02 | Std |  |
| 33.15 | 1238 | 1234 | *Cis*-neral | - | - | - | - | - | - | - | - | - | - | Std |  |
| 33.21 | 1245 | 1246 | *Cis*-3-hexenyl isovalerate | - | - | - | - | - | - | - | - | - | - | MS-RI |  |
| 34.03 | 1246 | 1248 | carvone | - | 1.34±0.22 | - | 1.04±0.09 | - | - | - | - | - | - | MS-RI |  |
| 34.28 | 1253 | 1255 | *Cis*-geraniol | 0.30±0.02 | - | 5.21±0.23 | - | - | 1.24±0.11 | 0.72±0.04 | 1.12±0.14 | - | - | Std |  |
| 34.32 | 1257 | 1256 | linalyl acetate | - | - | - | 0.07±0.01 | 0.29±0.02 | - | - | - | 0.65±0.04 | 1.52±0.23 | Std |  |
| 34.32 | 1267 | 1257 | geranial | - | - | - | - | - | - | - | 0.13±0.01 | - | - | Std |  |
| 36.31 | 1269 | 1263 | benzyl isobutyrate | - | - | - | - | - | - | - | 0.05±0.01 | - | - | MS-RI |  |
| 36.33 | 1270 | 1266 | α-citral | - | - | 0.03±0.01 | - | - | - | - | - | - | - | Std |  |
| 36.39 | 1285 | 1288 | anethole | - | - | 0.05±0.01 | - | - | - | - | - | - | - | Std |  |
| 36.42 | 1289 | 1290 | bornyl acetate | - | - | 0.02±0.01 | - | - | - | - | - | - | - | Std |  |
| 36.48 | 1298 | 1298 | *Trans*-pinocarvone acetate | - | 0.10±0.01 | 0.04±0.01 | 0.19±0.02 | - | - | - | 0.06±0.01 | - | 0.34±0.04 | MS-RI |  |
| 37.28 | 1325 | 1322 | methyl geraniate | - | 0.10±0.01 | - | - | - | - | - | - | - | 0.08±0.01 | MS-RI |  |
| 37.50 | 1327 | 1331 | myrtenyl acetate | - | 0.05±0.01 | 0.03±0.01 | 0.05±0.01 | - | 0.10±0.01 | 0.05±0.01 | - | 0.11±0.01 | 28.13±0.39 | MS-RI |  |
| 38.14 | 1343 | 1340 | exo-2-hydroxycineole acetate | 0.08±0.01 | 0.32±0.02 | 0.27±0.02 | 0.19±0.01 | 0.06±0.01 | 0.22±0.02 | 0.15±0.02 | - | 0.23±0.02 | - | MS-RI |  |
| 38.41 | 1349 | 1351 | α-terpinyl acetate | 0.54±0.02 | 1.17±0.14 | 2.64±0.19 |  | 0.82±0.07 | 1.20±0.11 | 0.37±0.02 | - | 1.97±0.16 | 0.39±0.02 | MS-RI |  |
| 38.64 | 1357 | 1357 | benzyl 2-methyl butanoate | - | 0.15±0.02 | 0.04±0.01 | 0.06±0.01 | 0.04±0.01 | - | - | 0.12±0.01 | - | 0.11±0.02 | MS-RI |  |
| 38.71 | 1359 | 1359 | eugenol | 0.06±0.01 | 0.11±0.02 | 1.33±0.04 | - | 0.07±0.01 | - | 0.16±0.02 | - | 0.14±0.02 | - | Std |  |
| 38.79 | 1365 | 1366 | neryl acetate | - | - | - | 0.10±0.02 | - | 0.19±0.01 | - | 0.06±0.01 | - | 0.35±0.02 | Std |  |
| 39.46 | 1381 | 1379 | geranyl acetate | 2.53±0.11 | 5.31±0.17 | 5.83±0.09 | 1.79±0.14 | 3.07±0.12 | 4.57±0.22 | 4.59±0.08 | 2.88±0.09 | 5.60±0.20 | 2.63±0.12 | Std |  |
| 39.57 | 1390 | 1388 | β-cubebene | - | - | - | - | - | - | - | 0.26±0.02 | - | - | Std |  |
| 40.07 | 1391 | 1395 | β-elemene | - | 0.09±0.01 | 0.25±0.03 | 0.07±0.01 | 0.15±0.01 | 0.56±0.10 | - | - | - | - | Std |  |
| 40.25 | 1404 | 1401 | methyleugenol | 1.76±0.05 | 3.01±0.03 | 2.91±0.14 | 2.51±0.04 | 0.68±0.02 | 2.16±0.02 | 2.10±0.09 | 1.72±0.02 | 2.64±0.03 | 2.33±0.02 | MS-RI |  |
| 41.19 | 1428 | 1430 | β-caryophyllene | 2.51±0.22 | 0.69±0.04 | 0.89±0.04 | 1.49±0.14 | 0.88±0.04 | 0.97±0.10 | 3.72±0.21 | 1.19±0.06 | 1.10±0.11 | 0.43±0.04 | Std |  |
| 41.36 | 1436 | 1431 | p-Mentha-1,8-dien-7-yl acetate | - | - | - | - | - | - | - | - | - | 0.12±0.02 | MS-RI |  |
| 41.47 | 1437 | 1434 | γ-elemene | - | - | - | - | - | - | 0.04±0.01 | - | - | - | Std |  |
| 41.76 | 1440 | 1437 | α-guajene | - | 0.06±0.01 | 0.02±0.01 | - | - | 0.03±0.01 | - | - | - | - | MS-RI |  |
| 42.20 | 1441 | 1443 | aromadendrene | - | 0.41±0.07 | 0.11±0.01 | - | - | 0.09±0.01 | 0.46±0.09 | 0.35±0.8 | 0.09±0.01 | - | Std |  |
| 42.29 | 1455 | 1456 | α-humulene | 1.25±0.10 | 1.11±0.12 | 2.85±0.16 | 0.91±0.06 | 0.41±0.04 | 0.55±0.09 | 0.78±0.06 | 0.72±0.03 | 0.57±0.03 | 1.47±0.09 | Std |  |
| 42.55 | 1460 | 1458 | alloaromadendrene | - | - | 0.13±0.01 | - | - | 0.10±0.02 | - | - | - | - | MS-RI |  |
| 42.86 | 1480 | 1480 | γ-muurolene | - | 0.03±0.01 | 0.12±0.01 | - | 0.07±0.01 | 0.21±0.03 | - | - | - | - | Std |  |
| 43.02 | 1485 | 1485 | germacrene D | - | 0.03±0.01 | - | - | - | - | - | - | - | - | Std |  |
| 43.09 | 1496 | 1490 | 2-tridecanone | - | 0.08±0.01 | 0.08±0.01 | 0.03±0.01 | 0.05±0.01 | 0.10±0.01 | 0.10±0.01 | 0.11±0.01 |  | 0.06±0.01 | MS-RI |  |
| 43.22 | 1496 | 1499 | isoeugenyl methyl ether | - | 0.05±0.01 | - | 0.06±0.01 | - | - | 0.07±0.01 | - | - | - | MS-RI |  |
| 43.28 | 1497 | 1497 | β-selinene | 0.09±0.01 | 0.10±0.02 | 0.39±0.03 | 0.08±0.01 | 0.25±0.02 | 0.64±0.02 | - | - | - | - | Std |  |
| 43.52 | 1498 | 1499 | α-selinene | - | 0.09±0.01 | 0.40±0.04 | 0.05±0.01 | 0.27±0.02 | 0.69±0.03 | 0.18±0.02 | - | - | - | Std |  |
| 43.55 | 1503 | 1506 | geranyl acetate | - | 0.26±0.02 | 0.03±0.01 | - | - | - | - | - | - | - | Std |  |
| 43.57 | 1506 | 1512 | β-bisabolene | - | - | - | - | - | - | - | - | - | - | MS-RI |  |
| 43.59 | 1492 | 1512 | γ-selinene | - | - | - | - | - | - | - | - | - | - | Std |  |
| 43.61 | 1514 | 1513 | neryl butyrate | - | - | - | - | - | - | - | - | - | - | MS-RI |  |
| 43.64 | 1515 | 1515 | geranyl isobutyrate | - | - | - | 0.34±0.03 | - | - | - | 0.31±0.02 | - | - | MS-RI |  |
| 43.66 | 1515 | 1515 | *Cis*-γ-bisabolene | - | - | 0.04±0.01 | - | - | - | - | - | - | - | MS-RI |  |
| 44.06 | 1521 | 1520 | dihydroeugenyl butanoate | 4.42±0.15 | 2.78±0.15 | 4.30±0.12 | 7.13±0.12 | 0.45±0.06 | 2.29±0.09 | 1.26±0.11 | 4.84±0.09 | 7.38±0.11 | 2.31±0.21 | MS-RI | [7] |
| 44.23 | 1523 | 1520 | δ-cadinene | 0.10±0.01 | - | 0.06±0.01 | - | - | 0.05±0.01 | - | - | - | - | MS-RI |  |
| 44.84 | 1536 | 1542 | elemol | - | 0.08±0.01 | - | - | - | 0.04±0.01 | 0.07±0.01 | 0.09±0.01 | - | - | MS-RI |  |
| 44.86 | 1547 | 1550 | selina-3,7(11)-diene | 0.12±0.01 |  |  | 0.09±0.01 | - | - | - | - | 0.19±0.02 | - | MS-RI |  |
| 45.12 | 1563 | 1563 | *Trans*-nerolidol | 0.06±0.01 | 0.07±0.01 | 0.06±0.01 | 0.03±0.01 | - | - | 0.05±0.01 | 0.07±0.01 | - | - | Std |  |
| 45.72 | 1572 | 1572 | caryophyllenyl alcohol | - | 0.03±0.01 | 0.05±0.01 | 0.08±0.01 | - | 0.08±0.01 | - | - | - | - | MS-RI |  |
| 45.87 | 1578 | 1579 | spathulenol | - | 0.28±0.02 | 0.45±0.04 | - | 0.04±0.01 | 0.26±0.02 | 0.11±0.01 | 0.07±0.01 | 0.04±0.01 | - | MS-RI |  |
| 45.97 | 1580 | 1581 | *Cis*-caryophyllene oxide | 0.03±0.01 | - | 0.02±0.01 | 0.08±0.01 | - | - | - | 0.09±0.01 | - | - | MS-RI |  |
| 46.11 | 1583 | 1583 | *Trans*-caryophyllene oxide | 0.16±0.01 | 0.53±0.04 | 0.48±0.02 | 0.52±0.04 | 0.09±0.01 | 0.24±0.02 | 1.11±0.10 | - | 0.22±0.02 | 0.10±0.01 | MS-RI |  |
| 46.31 | 1585 | 1589 | globulol | - | 0.04±0.01 | 0.21±0.02 | - | - | 0.09±0.01 | 0.05±0.01 | - | - | - | MS-RI |  |
| 46.46 | 1601 | 1600 | guaiol | 0.11±0.01 | 0.14±0.01 | - | 0.31±0.03 | - | 0.06±0.01 | 0.06±0.01 | 0.10±0.01 | - | 0.08±0.01 | Std |  |
| 46.71 | 1603 | 1603 | α-dihydro(10,11)bisabolol | 0.09±0.01 | 0.25±0.02 | - | - | - | - | 0.22±0.02 | 0.18±0.02 | - | - | MS-RI |  |
| 46.78 | 1606 | 1608 | humulene epoxide II | 0.14±0.01 | - | 0.53±0.02 | 0.55±0.02 | - | - | - | 0.47±0.04 | 0.17±0.01 | 0.25±0.02 | MS-RI | [8] |
| 46.75 | 1613 | 1617 | selina-6-en-4-ol | - | 0.34±0.04 | - | - | - | 0.11±0.01 | 0.25±0.02 | - | - | - | MS-RI |  |
| 46.98 | 1631 | 1631 | dihydroeugenyl pentanoate | 1.29±0.09 | 0.52±0.04 | 0.42±0.04 | 0.67±0.04 | 0.11±0.01 | 0.40±0.04 | 1.11±0.07 | 1.56±0.21 | 0.94±0.06 | 0.12±0.01 | MS-RI | [7] |
| 47.19 | 1637 | 1632 | *Cis*-cadin-4-en-7-ol | - | - | - | - | - | - | - | - | - | - | MS-RI | [4] |
| 47.24 | 1642 | 1634 | γ-eudesmol | 0.07±0.01 | - | 0.03±0.01 | 0.10±0.01 | - | - | 0.05±0.01 | 0.11±0.02 | 0.12±0.01 | 0.07±0.01 | Std |  |
| 47.31 | 1642 | 1638 | alloaromadendrene epoxide | - | 0.05±0.01 | 0.34±0.03 | - | - | - | 0.16±0.01 | - | - | - | MS-RI |  |
| 47.37 | 1642 | 1640 | epi-α-muurolol | - | 0.25±0.04 | - | 0.23±0.02 | - | 0.17±0.02 | - | 0.20±0.04 | - | - | MS-RI |  |
| 47.41 | 1643 | 1641 | caryophylla-4(12),8(13)-dien-5β-ol | 0.12±0.01 | - | - | - | - | - | 0.30±0.04 | - | - | - | MS-RI |  |
| 47.78 | 1652 | 1655 | α-cadinol | - | - | - | - | 0.11±0.01 | - | - | - | - | - | Std |  |
| 47.81 | 1656 | 1656 | neointermedeol | 0.08±0.01 | 0.15±0.02 | - | 0.06±0.01 | - | 0.70±0.04 | 0.18±0.02 | 0.08±0.01 | 0.15±0.02 | - | MS-RI | [9] |
| 47.87 | 1660 | 1657 | α-selinen-11-en-4-ol | 0.07±0.01 | 0.21±0.02 | - | 0.09±0.01 | - |  | 0.12±0.01 | 0.06±0.01 | 0.05±0.01 | - | MS-RI |  |
| 48.18 | 1663 | 1661 | epi-globulol | 0.07±0.01 | 0.07±0.01 | - | 0.05±0.01 | - | 0.06±0.01 | - | 0.06±0.01 | 0.05±0.01 | - | MS-RI |  |
| 48.32 | 1686 | 1675 | α-bisabolol | - | - | - | - | - | - | - | - | - | - | Std |  |
| 48.54 | 1682 | 1682 | ledene oxide (I) | - | - | - | - | - | - | - | - | - | - | MS-RI | [10’] |
| 48.90 | 1700 | 1713 | eudesm-7(11)-en-4-ol | 0.17±0.02 | 0.10±0.01 | 0.07±0.01 | 0.06±0.01 | - | 0.03±0.01 | 0.15±0.03 | 0.08±0.01 | 0.21±0.03 | - | MS-RI | [11] |
| 50.30 | 1760 | 1766 | benzyl benzoate | - | 0.11±0.01 | 0.09±0.01 | 0.04±0.01 | - | - | - | 0.17±0.01 | 0.08±0.01 | - | MS-RI |  |
|  |  |  |  |  |  |  |  |  |  |  |  |  |  |  |  |
|  |  |  | **Percentage of identified compounds** | **98.66** | **93.84** | **95.03** | **96.28** | **99.88** | **98.33** | **97.57** | **96.14** | **98.43** | **99.23** |  |  |
|  |  |  | **Number of identified compounds** | **48** | **68** | **71** | **63** | **45** | **58** | **49** | **52** | **45** | **39** |  |  |

Data are expressed in percentage (%). *ID = Identification methods. MS: by comparison of the mass spectrum with those of the computer mass libraries ADAMS, NIST11 and by interpretation of the mass spectra fragmentations. RI: by comparison of retention index with those reported in literature. Std: by comparison of the retention time and mass spectrum of available authentic standars. No-polar column ZB-5. Data are the mean of three replicatres ± standard deviation.

**REFERENCES OF SUPPLEMENTARY MATERIAL**

1. Ghasemi, E.; Yamini, Y.; Bahramifar, N.; Sefidkon, F. Comparative analysis of the oil and supercritical CO_2_ extract of Artemisia sieberi. ***J. Food Eng.*** 2007, *79(1),* 306-311.
2. Tiitinen, K.; Hakala, M.; Kallio, H. Headspace volatiles from frozen berries of sea buckthorn (*Hippophae rhamnoides* L.). *Eur Food Res Technol*. 2006, *223*, 455-460.
3. Zoghbi, M.G.B.; Andrade, E.H.A.; da Silva, M.H.; Maia, J.G.S.; Luz, A.I.R.; da Silva, J.D. Chemical variation in the essential oils of Hyptis crenata Pohl ex Benth. ***Flavour Fragr. J.*** 2002, *17(1)*, 5-8.
4. Su, Y.C.; Ho, C.L.; Wang, E.I.C.; Chang, S.T. Antifungal activities and chemical compositions of essential oils from leaves of four eucalypts. ***Taiwan J. For. Sci.*** 2006, *21(1)*, 49-61.
5. Högnadóttir, Á.; Rouseff, R.L. Identification of aroma active compounds in orange essence oil using gas chromatography-olfactometry and gas chromatography-mass spectrometry. ***J. Chromatogr. A***. 2003, *998(1-2)*, 201-211.
6. Wu, S.; Zorn, H.; Krings, U.; Berger, R.G. Volatiles from submerged and surface-cultured beefsteak fungus, Fistulina hepatica. ***Flavour Fragr. J.*** 2007, *22(1),* 53-60.
7. M. Usai, M. Mulas, M. Marchetti M, ‘Chemical compositions of essential oils of leaves and flowers from five cultivars of myrtle (Myrtus communis L.)’, J. Essent. Oil Res. 2015, 27(6), 465- 476.
8. Ouijano, C.E.; Salamanca, G.; Pino, J.A. Aroma volatile constituents of Colombian varieties of mango (Mangifera indica L.). ***Flavour Fragr. J.*** 2007, *22*, 401-406.
9. Lucero, M.E.; Fredrickson, E.L.; Estell, R.E.; Morrison, A.A.; Richman, D.B. Volatile composition of Gutierrezia sarothrae (Broom Snakeweed) as determined by steam distillation and solid phase microextraction. ***J. Essent. Oil Res*.** 2006, *18(2)*, 121-125.
10. Fakhari A.R.; Sonboli A.; Heydari R. Composition of the essential oil of Rhabdosciadium strausii from Iran*.* ***Chem. Nat. Compd*.** 2005, *41*, 413-414.
11. Ramírez, J.; Gilardoni, G; Ramón, E; Tosi, S; Picco, A.M.; Bicchi ,C; Vidari, G. Phytochemical study of the Ecuadorian species *Lepechinia mutica* (Benth.) Epling and high antifungal activity of carnosol against *Pyricularia oryzae*. *Pharmaceuticals*. 2018, *11,* 33-46.
